# Synthesis, molecular docking and antibacterial activity of an oxadiazole-based lipoteichoic acid inhibitor and its metabolites

**DOI:** 10.1016/j.molstruc.2023.134977

**Published:** 2023-04-15

**Authors:** Michaela Serpi, Fabrizio Pertusati, Chiara Morozzi, Giulia Novelli, Daniele Giannantonio, Katrina Duggan, Serena Vittorio, Ian A. Fallis, Laura De Luca, David Williams

**Affiliations:** a School of Chemistry, Cardiff University, Main Building, Park Place, Cardiff, Wales CF10 3AT, United Kingdom; b School of Pharmacy and Pharmaceutical Sciences, Redwood Building, King Edwards VII avenue, Cardiff, Wales CF10 3NB, United Kingdom; c Oral and Biomedical Sciences, School of Dentistry, Cardiff University, Cardiff, United Kingdom; d Department of Chemical, Biological, Pharmaceutical and Environmental Sciences, University of Messina, Viale F. Stagno D'Alcontres 31, Messina I-98125, Italy

## Abstract

Amongst drug resistant Gram-positive bacteria, *Staphylococcus aureus* is a pathogen of great concern as it is the leading cause of life-threatening nosocomial and community acquired infections which are often associated with implanted medical devices. The biosynthesis of lipotheicoic acid (LTA) by *S. aureus* has been recognized as a promising antibacterial target, owing its critical role in the growth and survival of Gram-positive bacteria. Here we report for the first time the chemical synthesis and characterisation of an oxadiazole based compound (1771), previously described as an inhibitor of LTA biosynthesis by targeting Lta synthase enzyme (LtaS). To investigate its controversial mode of action, we also performed molecular docking studies, which indicated that 1771 behaves as a competitive inhibitor against LtaS. We also synthesised and evaluated the antimicrobial activity of 1771 metabolites which we have identified from its decomposition in mouse serum, proving that the biological activity was caused by intact 1771.

## Highlights

• The full synthesis of compound 1771 was reported.
• Compound 1771 was found to have a good binding mode with LtaS active site reinforcing the hypothesis that 1771 could act as inhibitor of this enzyme to block the synthesis of LTAs.
• Three main metabolites were identified upon incubation of 1771 in mice serum (pH7.4) (half-life of 210 min).
• The lack of antibacterial activity of the synthesized metabolites indicated that the biological activity is caused by the intact compound 1771 rather than its fragments.

## Graphical abstract

**Figure:**
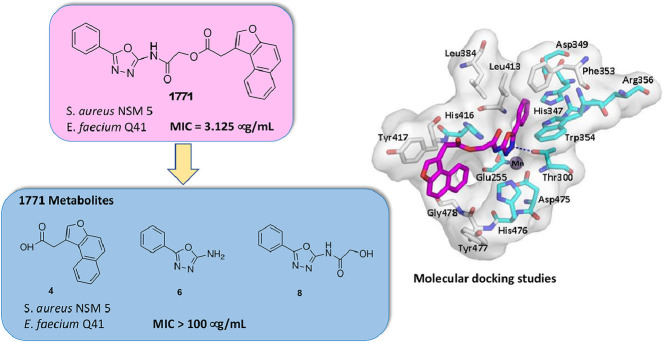

## 1 Introduction

There is rapid global emergence of Antimicrobial Resistance (AMR), which has led to the recurring failure of established antibiotics to treat and cure common infections. Without new antibiotics by 2050, it is estimated that AMR will cause 10,000,000 deaths per year with an associated $1 trillion global healthcare cost [1]. Amongst multi-drug resistant (MDR) Gram-positive bacteria, *Staphylococcus aureus* (methicillin-resistant and vancomycin-resistant *S. aureus* strains)[2,3] and vancomycin-resistant *Enterococcus faecium* (VRE *E. faecium*)[4] are leading causes of nosocomial infections, often involving the skin, soft tissue and urinary tract. Although two other antibiotics, namely, Daptomycin and Linezolid have been licensed to address systemic MDR *S. aureus*[5] and *E. faecium* infections [6], rapid resistance to these new drugs has occurred [7], [8], [9], [10]. Given this situation, the search for unexplored targets unique to Gram-positive bacteria, while critical for their growth and survival, is a promising strategy to design novel narrow-spectrum antibacterial agents with the potential to reduce spread of resistance across multiple bacterial species.

Techoic acids (TA) are glycopolymers present in the cell walls of Gram-positive bacteria and include wall teichoic acids (WTA) and lipoteichoic acids (LTA) [11], both of which play critical roles in many cellular processes [12], [13], [14], [15], [16], [17]. While WTA biosynthesis has been extensively investigated for design of new therapies to overcome resistant infections, only recently has LTA emerged as a promising novel antimicrobial target [18], [19], [20]. LTA, consists of a soluble polymer tethered to a membrane anchor facing the outer leaflet of the plasma membrane. There are five types of LTAs that are specific for bacteria. *Staphylococcus aureus* produces type I LTA, which is the most frequently encountered and best-characterized polymer [20]. Type I LTA is formed of alternating units of a polyglycerol joined via phosphodiester linkages and decorated with D-alanine and carbohydrate residues. The enzymatic machinery involved in *S. aureus* LTA biosynthesis has been identified. In particular, lipoteichoic acid synthase (LtaS) has been recognised as a key enzyme in LTA biosynthesis [21].

**Fig. 1:**
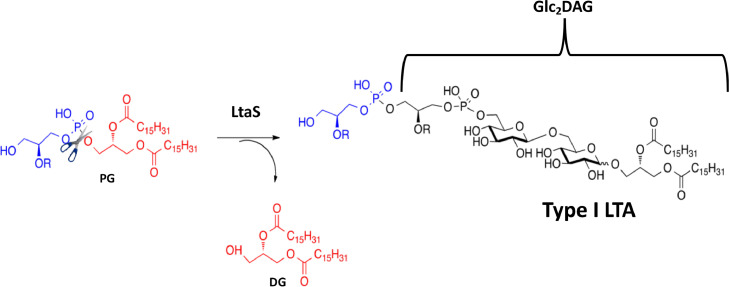
Chemical reaction for the biosynthesis of Type I LTA catalysed by LtaS.

To provide insight into the mechanism of LtaS, responsible for the polymerization of the glycerol phosphate chain, the entire eLtaS was crystallized and the structure solved with or without glycerol phosphate [21]. This investigation combined with mutagenesis studies resulted in a putative reaction mechanism for LtaS being proposed, while defining the active site residues essential for its function [22]. Specifically LtaS catalyzes the formation of the polyglycerolphosphate LTA backbone by hydrolyis of the glycerolphosphate head group of the membrane lipid phosphatidylglycerol (PG) and subsequent repeated addition of glycerol- phosphate units to diglucosyl-diacylglycerol (Glc_2_-DAG, see Fig. 1).

The absence of LtaS human homologues, the location of LtaS catalytic domain on the bacterial surface, and the essential requirement of LTA synthesis for bacterial growth and cell division, fulfill the key target features for development of new antibiotics against MDR Gram-positive bacteria.

**Fig. 2:**
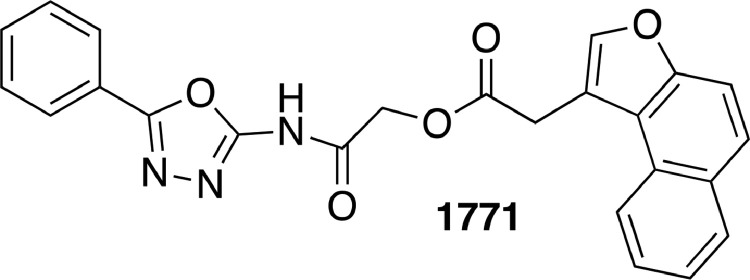
Structure of compound 1771.

LtaS has been validated as a target for development of antibiotics. In particular, a 1,3,4 oxadiazoyl based small molecule named 1771 (Fig. 2), has been shown to block LTA biosynthesis by *S. aureus* through binding to the active site of LtaS and preventing its association with glycerol phosphate [22]. The 1771 compound has been found to inhibit *in vitro* growth of meticillin resistant *S. aureus* (MRSA)[22] and vancomycin-resistant *E. faecium* (VRE) strains, to abrogate growth of MDR strains while displaying little or no cytotoxicity towards mammalian cells [23]. In a *S. aureus* sepsis mouse model, compound 1771, despite its significant instability in blood, demonstrated *in vivo* efficacy [22]. More recently, other 1,3,4 oxadiazoyl-based compounds were reported as potent inhibitors of *S. aureus* LTA biosynthesis [24,25]. Unexpectedly, a more recent study on the reconstitution of *S. aureus* LtaS activity fully disclaimed 1771 LtaS inhibition [26]. Intrigued by this controversy and conscious of the importance to identify new antibacterial leads, we decided to further investigate compound 1771. Compound 1771 was originally selected from screening of available molecules [22] and to the best of our knowledge, no rigorous synthesis has yet been reported. Herein, we disclose, for the first time the full chemical synthesis of compound 1771. Since this compound was previuosly found to be unstable in mouse, we considered the possibility that the reported activity might not only be due to the intact compound, but one of its metabolites. Therfore, once we identified the metabolites in mouse serum we synthesised them and evaluated their antimicrobial activity to assess their activity.

## 2 Experimental

### 2.1 General

All commercially available chemicals were supplied by Sigma-Aldrich or Fisher, and used without further purification. All solid reagents were dried for several hours under high vacuum prior to use. For analytical thin-layer chromatography (TLC), precoated aluminium-backed plates (60 F-54, 0.2 mm thickness; supplied by E. Merck AG, Darmstadt, Germany) were used and developed by an ascending elution method. After solvent evaporation, compounds were detected by quenching of fluorescence at 254 nm upon irradiation with a UV lamp. Column chromatography purifications were performed by automatic Biotage Isolera One. Fractions containing the product were identified by TLC, pooled and the solvent removed in vacuo. ^1^H and ^13^C NMR spectra were recorded on a Bruker Avance 500 spectrometer at 500 MHz and 125 MHz, respectively and auto-calibrated to the deuterated solvent reference peak. All ^13^C NMR spectra were proton-decoupled. Chemical shifts were given in parts per million (ppm) and coupling constants (*J*) measured in Hertz (Hz). The following abbreviations were used in the assignment of NMR signals: s (singlet), d (doublet), m (multiplet), and br (broad). The assignment of the signals was done based on the analysis of coupling constants and additional two-dimensional experiments (COSY, HSQC). Analytical High-Performance Liquid Chromatography (HPLC) analysis was performed using Spectra System SCM (with X-select-C18, 5 mm, 4.8 × 150 mm column), Varian Prostar system (LC Workstation- Varian Prostar 335 LC detector). All tested compounds showed >93% of purity by analytical HPLC. Low and high-resolution mass spectrometry were performed on a Bruker Daltonics MicroTof-LC system (atmospheric pressure ionization, electron spray mass spectroscopy) in positive or negative modes.

### 2.2 Chemistry

#### 2.2.1 Synthesis and characterization

2‑chloro-*N*-(5-phenyl-1,3,4-oxadiazol-2-yl)acetamide (**7**). To a suspension of 2-amino-5-phenyl-1,3,4-oxadiazole (**6**) (12.4 mmol, 2.00 g) in toluene (31 mL) at −25 °C, chloro acetyl chloride (26.04 mmol, 2.07 mL) was added dropwise. After 10 min, the resulting mixture was stirred at room temperature for 30 min. The reaction was then warmed to 65 °C and stirred for 3 h. The solvent was removed under vacuum giving 2-chloro-*N*-(5-phenyl-1,3,4-oxadiazol-2-yl)acetamide (**7**) as white powder (3.00 g, 99%) and used for the next step without any further purification. ^1^H NMR (500 MHz, DMSO‑d_6_): δ_H_ 12.27 (br, 1H, NH), 7.95 – 7.93 (m, 2H, CH-Ph), 7.64 – 7.61 (m, 3H, CH-Ph), 4.46 (s, 1H, CH_2_Cl); ^13^C NMR (125 MHz, DMSO-d_6_): δ_C_ 166.47 (CONH), 161.17 (C-2), 157.41 (C-5), 132.24 (CH-Ph), 129.95 (CH-Ph), 126.51 (CH-Ph), 123.77 (C-Ph), 43.54 (CH_2_).

2-oxo-2-((5-phenyl-1,3,4-oxadiazol-2-yl)amino)ethyl 2-(naphtho[2,1-*b*]furan-1-yl)acetate (**1**, compound 1771). To a mixture of **4** (1.3 mmol, 0.30 g) and **7** (1.4 mmol, 0.34 g) in DMF (1.43 mL), sodium iodide (0.13 mmol, 19 mg) and triethylamine (1.4 mmol, 199 mL) were added and the resulting mixture stirred at 90 °C for 3 h. The crude was then purified by chromatography giving the expected product (**1**) as a light yellow powder (0.305 g, 50% yield). *R_f_* : 0.23 (CH_2_Cl_2_/CH_3_OH 97:3); ^1^H NMR (500 MHz, CDCl_3_): δ_H_ 8.19 (d *J* = 8.2 Hz, 1H, CH-Ar), 7.93 – 7.88 (m, 3H, CH-Ar), 7.81 (s, 1H, CH-Ar), 7.69 – 7.67 (m, 1H, CH-Ar), 7.60 – 7.39 (m, 6H, CH-Ar), 4.92 (s, 2H, CH_2_), 4.26 (s, 2H, CH_2_). ^13^C NMR (125 MHz, DMSO): δ_C_ 176.15 (C=O ester), 170.40 (CONH), 160.70 (C, Oxadiazole), 157.22 (C, Oxadiazole), 152.67 (C-Ar), 143.85 (CH-Ar), 131.70 (CH-Ar), 130.31 (C-Ar), 129.46 (CH-Ar), 128.88 (CH-Ar), 127.76 (C-Ar), 126.58 (CH-Ar), 125.97 (CH-Ar), 125.79, (CH-Ar), 124.42 (CH-Ar), 123.33 (CH-Ar), 120.87 (C-Ar), 114.71 (C-Ar), 112.64 (CH-Ar), 62.88 (CH_2—_OCO), 30.35 (CH_2_). Reverse-phase HPLC, eluting with H_2_O/ ACN 40/60 for 25 min; to 0/100 in 5 min, Flow = 1 mL/min, λ = 263 nm, *t*_R_= 3.94 min (95%). HRMS: m/z calcd 428.1249 (M + H)^+^, found 428.1246 (M + H)^+^.

### 2.3 Stability studies

#### 2.3.1 Chemical stability of 1771 in phosphate buffer at pH 7.4

Compound **1** was incubated in 100 mM phosphate buffer at pH 7.4 at 37 °C in a water bath. The incubation mixture was prepared by dissolving 400 μL of a 1 mg/mL stock solution of compound **1** in DMSO in preheated buffer solutions (400 μL). Aliquots of 25 μL were withdrawn at appropriate intervals and analyzed by HPLC on a C18 column with a 0–100% 0.1% TFA H_2_O/MeOH gradient in 20 min at a flow rate of 1.0 mL/min.

#### 2.3.2 Stability in mouse serum

The incubation mixture for study of compound **1** stability in serum was prepared by dissolving 400 μL of 1 mg/mL stock solution in 260 μL of 100 mM phosphate buffer at pH 7.4 and 140 μL of mouse serum. The mixture was incubated at 37 °C in a water bath. Samples (25 μL) were drawn at appropriate intervals, mixed with methanol (25 μL) and centrifuged (5 min at 6000 rpm, RCF 2000 g)). Aliquots of 25 μL of the supernatant liquid were analyzed by HPLC on a C18 column with a 0–100% 0.1% TFA H_2_O/MeOH gradient in 20 min at a flow rate of 1.0 mL/min.

### 2.4 Molecular modeling studies

#### 2.4.1 Docking studies

The crystal structure of LtaS in complex with its substrate glycerol-phosphate was retrieved from the RCSB Protein Data Bank (PDB code 2w5s) [Lu, PNAS 2009]. The ligand and the water molecules were discarded, and hydrogens added using Discovery Studio 2.5.

The structures of the compounds were constructed using Discovery Studio 2.5 [Discovery Studio 2.5.5; Accelrys: San Diego, CA, 2009; http://www.accelrys.com]. The conformational behavior of the ligands was investigated by a MonteCarlo procedure (as implemented in the VEGA suite of programs that generated 1000 conformers by randomly rotating the rotors [27].

All obtained geometries were stored and optimized to avoid high-energy rotamers. The 1000 conformers were clustered by similarity to discard redundancies. In this analysis, two geometries were considered non-redundant when they differed by more than 60 in at least 2one torsion angle. For each derivative, the lowest energy structure was submitted to docking simulations.

Docking studies were undertaken using GOLD Suite 5.0.1. The region used by GOLD software was defined to contain the residues within 10 Å from the original position of the co-crystallized ligand. ChemPLP was chosen as a fitness function. The standard default settings were used in all calculations and the ligands submitted to 100 genetic algorithm runs. The highest GOLD fitness score was selected for analysis and representation. The docking protocol was validated by docking the native co-crystallized ligand glycerol-phosphate into LtaS active site. The comparison of the docking results with the co-crystallized conformation showed a good superimposition of the two forms resulting in RMSD value equal to 0.6493 Å, thus confirming the accuracy of our protocol in reproducing the experimental bound conformation.

After docking simulation, the obtained ligand-protein complex was optimized running 1000 steps energy minimization by means of NAMD, keeping the manganese ion fixed and applying harmonic constraints to Thr300, Glu255, Asp475 and His476 involved in the coordination of the metal. The putative ligand-protein interactions were analyzed by using Discovery Studio.

#### 2.4.2 Molecular dynamic simulation

Classical MD simulation was performed using the NAMD 2.12 package [28] and the conformation of derivative 1771 docked into LtaS active site as an initial protein-ligand complex. The web-based graphical user interface CHARMM-GUI (http://www.charmm-gui.org/) [29] was used to set up the simulation and generate the initial input files. The manganese ion was replaced by zinc ion as there were no parameters for manganese ion in CHARMM 36 employed as a force field. The system was solvated in a cubic box of TIP3 water model and neutralized with KCl setting a salt concentration of 0.15 M. After 10 ps minimization by a conjugate gradient method, 500 ps equilibration in NVT ensemble was performed using Langevin dynamic at 310 K. The system was eventually simulated for 10 ns in NPT ensemble keeping the pressure around 1 atm. Harmonic restraints were applied to the zinc ion and the residues involved in metal coordination, Thr300, Glu255, Asp475, and His476. The time step was set to 2 fs and coordinates saved every 1 ps. Visualization and analysis of the trajectory were performed using VMD software [30]. The stability of the protein-ligand complex was evaluated by computing root-mean-square deviations for the protein backbone and the ligand using the “RMSD Trajectory Tool” implemented in VMD.

### 2.5 Biological evaluation

#### 2.5.1 Bacterial strains

A total of six bacterial strains were used in this study. These strains included clinical isolates from indwelling catheters of patients undergoing long-term catheterization in the Cardiff (Wales, UK) health region. *Staphylococcus aureus* NSM 5 [31]. *S. aureus* P10 6/9 [32]. *S. aureus* N49, and the vancomycin-resistant enterococcal (VRE) strains of *E. faecium* Q41, *E. faecium* M49 and *E. faecium* Q11 were isolated from patients from the Intensive Care Unit and hematology ward of University Hospital of Wales (UHW) in Cardiff (Wales, UK) [33].

#### 2.5.2 Preparation of bacterial suspensions

Frozen stock bacterial cultures (*S. aureus* NSM 5, *S. aureus* N49, *S. aureus* P10 6/9, *E. faecium* Q41, *E. faecium* M49, *E. faecium* Q11) were thawed and streaked on to blood agar and incubated aerobically overnight at 37 °C. After 24 h, 3–4 bacterial colonies were inoculated into 10 mL of sterile Muller-Hinton broth (MHB) and incubated overnight at 37 °C. The resulting bacterial growth was adjusted to a 0.5 McFarland Standard (approximately 10^8^ cells mL^−1^) using sterile MHB and then diluted a further 100-fold in MHB.

#### 2.5.2 Determination of minimum inhibitory concentrations (MICs) and measurement of bacterial viability (Alamar blue test)

A bacterial suspension (100 μL) was added to the wells of a 96-well microtitre plate. A 100 μL volume of the assessed compound was added to the assay plates and mixed with the bacteria by repeat pipetting. The compound was added at twice the required final working concentration to account for dilution. Positive control wells contained 200 μL of bacterial suspension only, and negative control wells only contained 200 μL of MHB. All microtitre plates were incubated at 37 °C for 12 h. All tests were repeated in triplicate and bacterial growth was determined by measuring the change in OD at 620 nm using a MicroPlate Reader (FLUOstar Omega). The percentage inhibition of bacterial growth was calculated using the following equation:

$$\begin{array}{ccc} \%inhibition & = & sample\;OD\;\left(620\;mn\right)-negative\;control\;OD\;\left(620nm\right)/\\ & & \;positive\;control\;OD\left(620\;nm\right)\times 100 \end{array}$$

The MIC was defined as the lowest concentration of antimicrobial agent which resulted in 80% or more reduction in absorbance compared to antimicrobial-free controls. Subsequently, 30 mL resazurin (0,01% solution) was added to all wells and incubated at 37 °C for another 2 h. Changes of color were observed and recorded.

#### 2.5.3 Determination of minimum biocidal concentrations (MBC)

Following determination of the MIC, the entire 200 μL volume of the incubated bacteria/drug suspension was transferred to 2 ml of fresh MHB (in triplicate) and incubated at 37 °C for 12 h. Negative and positive control samples were also prepared. The concentration where no visible growth occurred after incubation was recorded as the minimum biocidal concentration (MBC).

## 3 Results and discussion

### 3.1 Molecular modeling studies

**Fig. 3:**
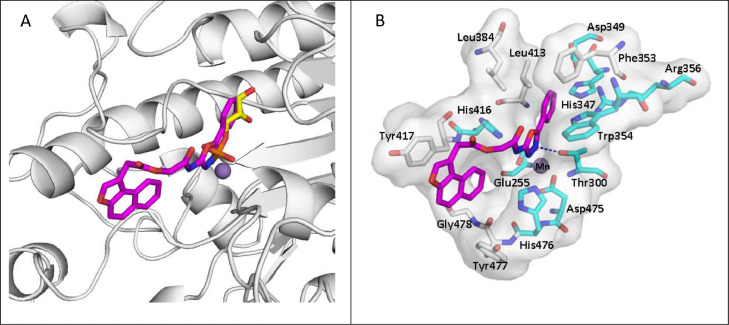
(A) Superimposition of glycerol phosphate (yellow sticks) and compound 1771 (magenta sticks) into the LtaS catalytic pocket. B) Proposed binding mode of compound 1771 (magenta sticks) into the LtaS active site. In cyan are highlighted the aminoacids residues that are reported to be crucial for LtaS enzyme activity. The manganese ion is represented by a violet sphere. The figure was created by using PyMOL software [The PyMOL Molecular Graphics System, Version 2.0 Schrödinger, LLC.].

Docking studies were performed to investigate the ability of compound 1771 to occupy the active site of LtaS by means of GOLD software, using the structure of LtaS co-crystallized with its substrate glycerol-phosphate (PDB code 2w5s). In Fig. 3, is a plausible binding mode for compound 1771 in the LtaS catalytic pocket is reported.

Of note is that the superposition of the docking result of compound 1771 (magenta) and the crystallographic pose of glycerol-phosphate (yellow) shows that the phenyl-1,3,4-oxadiazol portion of 1771 fits into the same region of the substrate glycerol phosphate (Fig. 3A), with one of the oxadiazole nitrogens overlapping the oxygen of the phosphate group involved in the coordination of the manganese ion. In the x-ray, the carbon chain of the substrate is located at the end of the pocket and elicits, through its hydroxyl groups, a network of hydrogen bonds with the surrounding residues His347,Asp349 and Arg356 [21]. The docking results suggest that the same region might be occupied by the phenyl moiety of 1771 which is involved in π-π interactions with Phe353 and π-cation interaction with Arg356. In addition, an H-bond interaction was detected between the nitrogen in position 3 of the oxadiazole ring of 1771 and the catalytic and metal-binding Thr300. Furthermore, the naphtho[2,1-b]furan moiety engages π-stacking interaction with Tyr417 (Fig. 3**B**). Some of the residues involved in the binding with compound 1771, such as Thr300 and Arg356, have been reported to be essential amino acids for LtaS function [21].

**Fig. 4:**
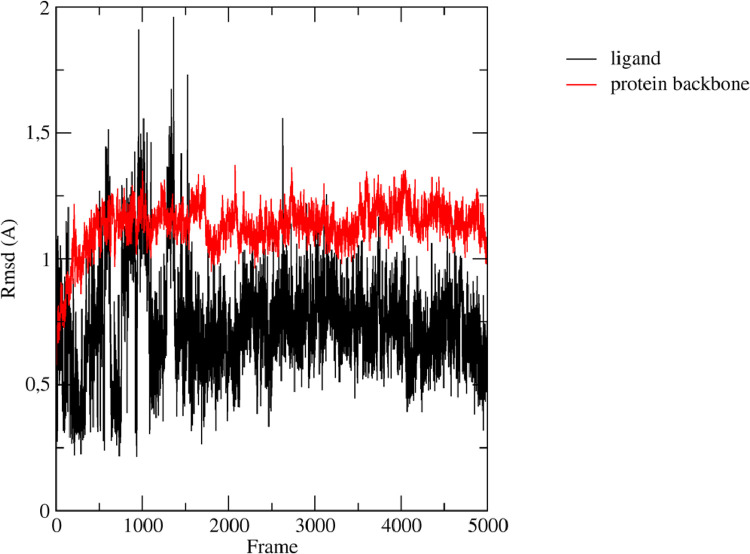
RMSD plot of the ligand (black) and LtaS protein backbone (red).

To assess stability of the predicted binding position, we performed 10 ns of MD simulation by means of NAMD software [28]. In Fig. 4, the root mean square deviation (RMSD) plot related to the protein backbone (in red) and the ligand (in black) is reported. The protein backbone RMSD converged after 1 ns (frame 500), remaining stable for the rest of the simulation. Similarly, after initial fluctuations, the ligand showed a stable behavior throughout the simulation. These observations highlight that compound 1771 forms a stable complex with LtaS reinforcing the hypothesis that 1771 could bind to LtaS active site inhibiting its enzymatic activity, as reported by Ritcher et al. [22]. During preparation of this manuscript, a study with an alternative computational of 1771 was published by Chee Wezen et al. [34]. The outcomes of this study revealed that 1771 might bind the LtaS active site with a slightly different orientation to that obtained in our docking model. In more details, the binding mode proposed by Wezen and co-workers suggested that the amide carbonyl oxygen of 1771 might be implicated in the coordination of the metal center. In our model, the same role might be assumed by the nitrogen in 3 of the oxadiazole ring. In the study of Wezen et al., the oxadiazole moiety si located in the region of the pocket lined by Arg356, His347 and His253 which correspond to the position occupied by the phenyl ring in our model. Instead, in both docking models the naphtho[2,1-b]furan portion is situated in the same aerea of the active site. Overall, both *in silico* investigations highlighted that 1771 might behave as a competitive inhibitor of LtaS and emphasizes the key role of the oxadiazole ring in the ligand-protein recognition process in good agreement with the experimental studies according to which this moiety is crucial for the activity [22].

### 3.2 Chemistry

**Scheme 1:**
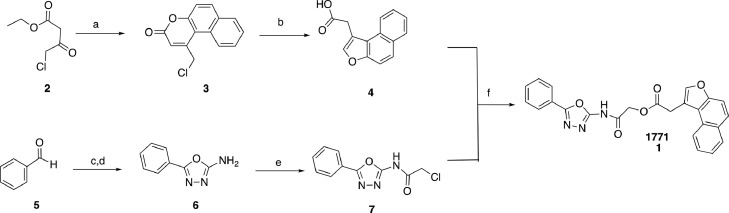
Synthesis of 1771. *Reagents and conditions:* (a) naphthalen-2-ol, H_2_SO_4_ conc., 5 °C, 24 h, 96%; (b) 1 M NaOH, reflux, 4 h, 89%; (c) Semicarbazide hydrochloride, sodium acetate, CH_3_OH, rt, 20 min, quant; (d) K_2_CO_3_, I_2_, 1,4-dioxane, 80 ºC, 48 h, 59% over two steps; (f) Chloro acetylchloride, toluene, −25 ºC to rt, 30 min and 60 ºC, 12 h, quant; (f) NaI, Et_3_N, DMF, 90 ºC, 3 h, 50%.

Scheme 1 depicts the convergent synthetic strategy we have devised for compound 1771 (**1**). Transesterification of ethyl 4-chloro-3-oxobutanoate **2** with naphthalen-2-ol, after Peachman rearrangement yielded the desired chlorocoumarine **3** (angular isomer) in excellent yield (96%) and as the only regioisomer [35,36] This was in agreement with literature data indicating that under this condition no other isomer was formed [37]. Compound **3** under basic conditions underwent a rearrangement to give the naphtho[2,1-*b*]furan acetic acid **4** in good (78%) yield [38]. With the first component of the compound 1771 in hand, we prepared the 2-amino-5-phenyl-1,3,4-oxadiazole **6** by condensation of benzaldehyde (**5**) with semicarbazide hydrochloride, followed by iodine mediated oxidative cyclization to afford **6** in 59% yield [39]. Subsequently, the desired oxadiazole derivative **7** was obtained by reaction of the aminodiazole **6** with chloroacetylchloride[40] in toluene in quantitative yield. The final step in the synthesis of 1771 was accomplished by coupling the two bulding blocks (**4** and **7**) in the presence of catalytic amount of sodium iodide and in presence of triethylamine in DMF [41].

**Fig. 5:**
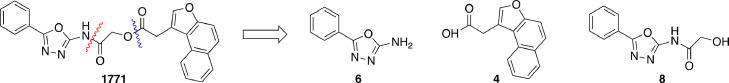
Degradation pathways for compound 1771 in mouse serum (pH 7.4) at 37 ºC leading to metabolites 6, 4 and 8.

**Fig. 6:**
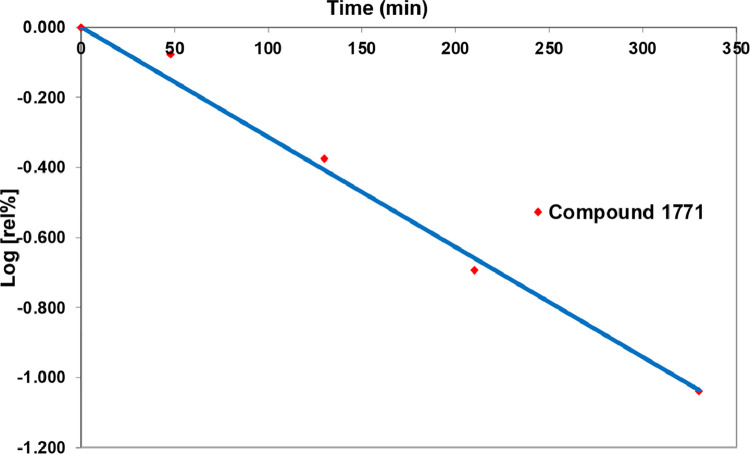
Stability studies of compound 1771 in mouse serum (pH 7.4) at 37 ºC.

*In vivo* experiments have shown that compound 1771 was highly unstable (*vide supra*). Analysis of mouse serum injected with a single dose of the drug was reported to show after 3 to 6 h, the presence of two fragments generated by the cleavage of 1771 [22]. However in this study, the structures of these two fragments were not reported, nor was their *in vitro* antibacterial activity. Since the ester and amide moieties are likely to be susceptible to hydrolysis under physiological conditions, we anticipated a degradation pathway for compound 1771 leading to three breakdown products. We have then confimed this pathway after incubation of 1771 in mouse serum at 37 °C by HPLC analysis following the formation of compounds **6, 4** and **8** over the time. (Figs. 5, S1 and S2). In this condition the enzymatic hydrolysis of 1771 followed pseudo-first-order kinetics with an half-life of 210 min (Fig. 6).

To assess the enzymatic stability, the rate of disappearance of 1771 was determined in mouse serum (pH 7.4) at 37 °C.

Having established the hydrolysis pathway for 1771 in mouse serum, we investigated the antibacterial activity of these fragments to establish if 1771 was the only active antibacterial agent or if growth inhibition also arose from the activity of these metabolites.

**Scheme 2:**
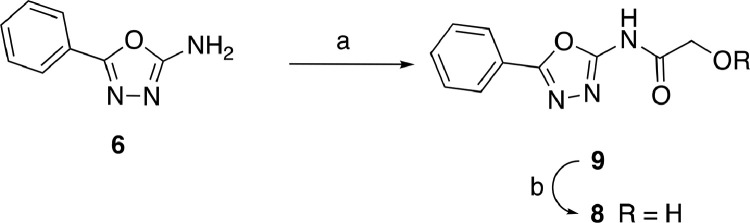
Synthesis of metabolite 8. *Reagents and conditions:* Acetoxy acetylchloride, toluene, −25 ºC to rt, 30 min and 60 ºC, 12 h, 80%; (b) Cesium formate, dry CH_3_OH, reflux, 5 h, 44%.

Naphtho[2,1-*b*]furan acetic acid **4** and the amino oxadiazole **6** were intermediates of the synthesis of compound 1771. Compound **8** was prepared according to the procedure reported in Scheme 2. Reaction of the aminooxadiazole **6** with acetoxy acetylchloride [40] in toluene followed by hydrolysis with cesium formate in methanolic solution[42] afforded the desired oxadiazole alcohol derivative **8** in 35% yield over two steps.

### 3.3 Antimicrobial activity

Antimicrobial activity of compound 1771 (**1**) and the metabolites **4, 6**, and **8** were tested against three different strains of *S. aureus* (NSM 5, N49, P10 6/9) as well as *E. faecium* (Q41, M49, Q11). Compound 1771 showed bacterial growth inhibition against all bacterial strains with MICs in the range of 3.12–6.25 μg/mL (Table 1). The minimum bactericidal concentration (MBC) were also calculated and the values reported in Table 1. MBCs for compound **1** were 50 μg/mL agaist both *S. aureus* NSM 5 and *E. faecium* Q41 and 100 μg/mL against the other tested strains. These results are in agreement with those reported in the litterature [22,23]. No microbial growth inhibition was observed with the three metabolite fragments **4, 6**, and **8** for all tested bacteria up to a concentration of 100 μg/mL indicating that the biological activity was caused by intact 1771, rather than its metabolites.

**Table 1:** *In vitro* antibacterial activities of 1771 (**1**) and compounds **4, 6** and **8**.

	MIC (MBC) μg/mL				
**Bacteria strains**	Reference or strains type	1771 (1)	4	6	8
*S. aureus* NSM 5	Ref. [31]	3.125 (50)	>100	>100	>100
*S. aureus* N49	Clinical isolate	6.25 (100)	>100	>100	>100
*S. aureus* P10 6/9	Ref. [32]	6.25 (100)	>100	>100	>100
*E. faecium* Q41	Clinical isolate	3.125 (50)	>100	>100	>100
*E. faecium* M49	Clinical isolate	6.25 (100)	>100	>100	>100
*E. faecium* Q11	Clinical isolate	3.125 (100)	>100	>100	>100

## 4 Conclusion

In conclusion, we have reported for the first time, the synthesis of the promising antibacterial agent compound 1771. Our molecular modeling and dynamic studies support the hypothesis that 1771 acts as an inhibitor of LtaS and reinforces the importance of the oxadiazole moiety for this activity. We also have proved the hydrolysis pathway of 1771 in mouse serum at 37 ºC with *t*_1/2_ of 210 min, identifying the three major breakdown products. We have chemically prepared these metabolites and shown that they are not responsible for 1771 antibacterial activity.Taken altogether, these results indicate that compound 1771 warrants further investigation as novel antibacterial agent against multi drug-resistant Gram-positive bacteria.

Guided by our molecular modeling studies, we are currently using the described synthetic strategy for the preparation of other derivatives aimed to improve stabilty of 1771 under physiological conditions, while also enhancing its antimicrobial activity. The results of these studies will be reported in due course.

## Supplementary material

Supplementary material associated with this article can be found, in the online version.

It includes intermediate compounds **3, 4, 6** and metabolite **8** characterization description; Fig. S1 with the stack of the HPLC traces of compounds **6, 8, 4** and 1771; Fig. S2 with the reverse-phase HPLC trace after 5h30 min incubation of 1771 in mouse serum (100 mM phosphate buffer pH 7.4) at 37 ºC copies of ^1^H, ^13^C -NMR spectra of compounds **1, 3, 4, 6, 7, 8** and **9**; HPLC traces and HR-MS for the final compounds **1** and metabolites **4, 6** and **8**.

## CRediT authorship contribution statement

**Michaela Serpi:** Supervision, Conceptualization, Writing – review & editing. **Fabrizio Pertusati:** Supervision, Conceptualization, Writing – review & editing. **Chiara Morozzi:** Investigation, Data curation. **Giulia Novelli:** Investigation. **Daniele Giannantonio:** Investigation. **Katrina Duggan:** Investigation. **Serena Vittorio:** Investigation. **Ian A. Fallis:** Supervision, Conceptualization, Writing – review & editing. **Laura De Luca:** Supervision, Conceptualization, Writing – review & editing. **David Williams:** Supervision, Conceptualization, Writing – review & editing.

## Declaration of Competing Interest

The authors declare that they have no known competing financial interests or personal relationships that could have appeared to influence the work reported in this paper.

## Data Availability

Data will be made available on request.

## Appendix Supplementary materials

Image, application 1
