# Supplementary material for: Synthesis, molecular docking and antibacterial activity of an oxadiazole-based lipoteichoic acid inhibitor and its metabolites
Source: J Mol Struct. 2023 Apr 15;1278:None. doi: 10.1016/j.molstruc.2023.134977 (PMC10836577; doi:10.1016/j.molstruc.2023.134977)
Supplement: Supplementary file 1 [file mmc1.pdf]

# Supporting Information

**Synthesis, molecular docking and antibacterial activity of an oxadiazole-based lipoteichoic inhibitor and its metabolites**

Michaela Serpi*[1], Fabrizio Pertusati[2], Chiara Morozzi,[2] Giulia Novelli[2], Daniele Giannantonio,[2] Katrina Duggan[3], Serena Vittorio[4], Jan Fallis[1], Laura De Luca[4], David Williams[3]

[1]School of Chemistry, Cardiff University Main Building, Park Place, CF10 3AT Cardiff, Wales, United Kingdom.

[2]School of Pharmacy and Pharmaceutical Sciences, Redwood Building, King Edwards VII avenue, CF10 3NB, Cardiff, Wales, United Kingdom.

[3]Oral and Biomedical Sciences, School of Dentistry, Cardiff University, Cardiff, UK

[4]Department of Chemical, Biological, Pharmaceutical, and Environmental Sciences, Polo Universitario SS. Annunziata, University of Messina, Viale Palatucci 13, I-98168, Messina, Italy

**General**

All commercially available chemicals were supplied by Sigma-Aldrich or Fisher and used without further purification. All solid reagents were dried for several hours under high vacuum prior to use. For analytical thin-layer chromatography (TLC), precoated aluminium-backed plates (60 F-54, 0.2 mm thickness; supplied by E. Merck AG, Darmstadt, Germany) were used and developed by an ascending elution method. After solvent evaporation, compounds were detected by quenching of fluorescence at 254 nm upon irradiation with a UV lamp. Column chromatography purifications were performed by automatic Biotage Isolera One. Fractions containing the product were identified by TLC, pooled and the solvent removed in vacuo. $^{1}H$ and $^{13}C$ NMR spectra were recorded on a Bruker Avance 500 spectrometer at 500 MHz and 125 MHz, respectively and auto-calibrated to the deuterated solvent reference peak. All $^{13}C$ NMR spectra were proton-decoupled. Chemical shifts were given in parts per million (ppm) and coupling constants (*J*) measured in Hertz (Hz). The following abbreviations were used in the assignment of NMR signals: s (singlet), d (doublet), m (multiplet), and br (broad). The assignment of the signals was done based on the analysis of coupling constants and additional two-dimensional experiments (COSY, HSQC). Analytical High-Performance Liquid Chromatography (HPLC) analysis was performed using Spectra System SCM (with X-select-C18, 5 mm, 4.8 x 150 mm column), Varian Prostar system (LC Workstation- Varian Prostar 335 LC detector). All tested compounds showed >93 % of purity by analytical HPLC. Low and high-resolution mass spectrometry were performed on a Bruker Daltonics MicroTof-LC system (atmospheric pressure ionization, electron spray mass spectroscopy) in positive or negative modes.

**1-(chloromethyl)-3H-benzo[f]chromen-3-one (3)**. To a suspension of naphthalen-2-ol (8.77 g, 61 mmol) and ethyl-4-chloroacetoacetate (8.21 mL, 61 mmol), conc. $H_2SO_4$ (6.50 mL, 122 mmol) was added dropwise at 0 °C. The system became a yellow-orange suspension. The reaction mixture was stirred at 0 °C for 24 h and then poured into ice-water (40 mL). The solid was filtered, washed with water and dried overnight on a high vacuum pump to afford 1-(chloromethyl)-3*H*-benzo[*f*]chromen-3-one (**3**) as a yellow powder (14.25 g, 96 %). $R_f$ : 0.31 (Hexane:EtOAc 7:3).$^{1}H$ NMR (500 MHz, DMSO-$d_6$): $\delta_H$ 8.55 (d, *J* = 8.9 Hz, 1H, C<u>H</u>-Ar), 8.25 (d, *J* = 8.9 Hz 1H, C<u>H</u>-Ar), 8.09 (dd, *J* = 8.1 and 1.4 Hz, 1H, C<u>H</u>-Ar), 7.78 - 7.74 (m, 1H, C<u>H</u>-Ar), 7.66 – 7.63 (m, 1H, C<u>H</u>-Ar), 7.60 (d, *J* = 8.9 Hz, 1H, C<u>H</u>-Ar), 6.88 (s, 1H, C<u>H</u>-Ar), 5.41 (s, 2H, C$\underline{H_2}$); $^{13}C$ NMR (125 MHz, DMSO-$d_6$): $\delta_C$ 159.17 (<u>C</u>-3), 154.67 (<u>C</u>-1), 151.86 (<u>C</u>-4a),

134.42 (CH-6), 130.89 (C-10a), 129.57 (CH-10), 128.39 (CH-9), 128.30 (C-6a), 125.73 (CH-8), 125.49 (CH-7), 117.53 (CH-5), 116.99 (CH-2), 111.89 (C-10b), 46.24 ($CH_2$).

**2-(naphthol[2,1-b]furan-1-yl)acetic acid (4)**. 1-(chloromethyl)-3*H*-benzo[*f*]chromen-3-one (**3**) (5 g, 20 mmol) was suspended at room temperature in a 1 M NaOH solution (192 mL). The mixture was refluxed for 4 h until the complete reaction was observed by TLC ($CH_2Cl_2$: $CH_3OH$ 94:6). A 1 M HCl solution was then added dropwise at 0 °C until pH 2. The system became a light-yellow suspension, which was filtered and the solid washed with 1M HCl (30 mL) and $H_2O$ (3 × 30 mL). The solid obtained was collected and dried overnight on high vacuum pump to afford the corresponding 2-(naphthol[2,1-*b*]furan-1-yl)acetic acid (**4**) as a light-yellow powder (3.59 g, 78 %). $R_f$ : 0.31 ($CH_2Cl_2$:$CH_3OH$ 94:6). $^1$H NMR (500 MHz, DMSO-d$_6$): $\delta_H$ 12.62 (br, 1H, $CO_2H$), 8.18 (d, $J$ = 8.5 Hz, 1H, CH-Ar), 8.05 (d, $J$ = 8.5 Hz, 1H, CH-Ar), 8.02 (s, 1H, CH-Ar), 7.84 (d, $J$ = 8.9 Hz, 1H, CH-Ar), 7.78 (d, $J$ = 8.9 Hz 1H, CH-Ar), 7.62-7.60 (m, 1H, CH-Ar), 7.53-7.50 (m, 1H, CH-Ar), 4.06 (s, 2H, $CH_2$); $^{13}$C NMR (125 MHz, DMSO-d$_6$): $\delta_C$ 172.17 ($CO_2H$), 152.61 (C-3a), 143.57 (CH-2), 130.29 (C-9b), 128.91 (CH-6), 127.88 (C-5a), 126.39 (CH-8), 125.58 (CH-7), 124.33 (CH-9), 123.03 (CH-5), 121.07 (C-9a), 115.73 (CH-1), 112.64 (CH-4) 30.89 ($CH_2$). HPLC, eluting with 0.1% TFA $H_2O$/MeOH 10:90 to 0/100 in 20 min, 0/100 for 5 min; Flow = 1 mL/min, λ= 291 nm $t_R$= 19.0 min (99%). HRMS (ESI; negative ion mode): *m*/*z* calcd 225.0554 (M - H)$^+$, found 225.0552 (M - H)$^+$.

**2-amino-5-phenyl-1,3,4-oxadiazole (6)**. To a stirred solution of semicarbazide hydrochloride (1.05 g, 9.5 mmol) and sodium acetate (0.78 g, 9.50 mmol) in water (19 mL), a solution of benzaldehyde **5** (0.97 mL, 9.50mmol) in methanol (19 mL) was added. The system become a white suspension which was stirred at room temperature for 20 min. The solvents were evaporated under reduced pressure and the residue dried for 2 h on a high vacuum pump. The resulting white powder was dissolved in 1,4-dioxane (94 mL) followed by the addition of anhydrous $K_2CO_3$ (3.93 g, 28.5 mmol) and iodine (2.9 g, 11.4 mmol) in sequence. The system became a purple-brown suspension. The reaction mixture was stirred at 95 °C for 3 h and then, after cooling at room temperature, additional $K_2CO_3$ anhydrous (1.97 g, 14.25 mmol) was added. The reaction mixture was stirred at 95 °C until complete conversion was observed by TLC ($CH_2Cl_2$:$CH_3OH$ 9:1). After being cooled to room temperature, it was treated with 5% $Na_2S_2O_3$ (w/v; 100 mL) and extracted with a mixture of $CH_2Cl_2$ and $CH_3OH$ 9:1 (4 × 80 mL). The combined organic layers were dried over anhydrous $Mg_2SO_4$ and concentrated. The given

residue was purified through silica gel column chromatography Biotage Isolera System using a gradient of $CH_3OH$ (2% to 10%) in $CH_2Cl_2$ as eluents to afford 2-amino-5-phenyl-1,3,4-oxadiazole (**6**) as a light-yellow powder (0.89 g, 59 %). $R_f$ : 0.32 ($CH_2Cl_2$/$CH_3OH$ 96:4). $^1H$ NMR (500 MHz, DMSO-$d_6$): $\delta_H$ 7.81 – 7.80 (m, 2H, C$\underline{H}$-Ph), 7.56 – 7.50 (m, 3H, C$\underline{H}$-Ph), 7.25 (br, 2H, N$\underline{H_2}$); $^{13}C$ NMR (125 MHz, DMSO-$d_6$): $\delta_C$ 164.35 ($\underline{C}$-2), 157.80 ($\underline{C}$-5), 130.82 ($\underline{C}$H-Ph), 129.69 ($\underline{C}$H-Ph), 125.49 ($\underline{C}$H-Ph), 124.87 ($\underline{C}$-Ph). HPLC, eluting with 0.1% TFA $H_2O$/MeOH 10:90 to 0/100 in 20 min, 0/100 for 5 min; Flow = 1 mL/min, $\lambda$= 272 nm $t_R$= 14.2 min (94 %). HRMS (ESI; positive ion mode): *m*/*z* calcd 162.0667 $(M + H)^+$, found 162.0670 $(M + H)^+$.

**2-oxo-2-((5-phenyl-1,3,4-oxadiazol-2-yl)amino)ethyl acetate (9)**. To a suspension of 2-amino-5-phenyl-1,3,4-oxadiazole (**6**) (1 g, 6 mmol) in dry toluene (20 mL) at -25 °C, acetoxyacetyl chloride (1 mL, 9 mmol) was added dropwise in 5 minutes. The system remained a white suspension. After 10 min the reaction mixture was allowed to go to room temperature and stirred at this temperature for 30 min, followed by an additional 12 h at 65°C. The reaction was then monitored by TLC ($CH_2Cl_2$:$CH_3OH$ 96:4) and no complete conversion observed. It was cooled again at -25°C again and another portion of acetoxyacetyl chloride (0.47 mL, 4 mmol) added, then it was allowed to reach room temperature and stirred at this temperature for 30 min followed by additional 12 h at 65°C until the reaction was completed. The solvent was removed under vacuum and the residue was purified through silica gel flash column chromatography using a gradient of $CH_3OH$ in $CH_2Cl_2$ (2% to 10%) as eluents to afford 2-oxo-2-((5-phenyl-1,3,4-oxadiazol-2-yl)amino)ethyl acetate (**9**) as a white powder (1.30 g, 80 %). $R_f$: 0.42 ($CH_2Cl_2$/$CH_3OH$ 96:4). $^1H$ NMR (500 MHz, $CDCl_3$): $\delta_H$ 11.11 (br, 1H, N$\underline{H}$), 8.04-8.02 (m, 2H, $\underline{C}$H-Ph), 7.55 - 7.49 (m, 3H, $\underline{C}$H-Ph), 4.90 (s, 2H, C$\underline{H_2}$), 2.25 (s, 3*H*, C$\underline{H_3}$); $^{13}C$ NMR (125 MHz, DMSO-$d_6$): $\delta_C$ 169.96 ($\underline{C}ONH_2$ and $\underline{C}$-2), 160.61 ($\underline{C}O_2CH_2$), 159.96 ($\underline{C}$-5), 131.68 ($\underline{C}$H-Ph), 129.42 ($\underline{C}$H-Ph), 125.97 ($\underline{C}$H-Ph), 123.27 ($\underline{C}$-Ph), 62.24 ($\underline{C}H_2$), 20.31 ($\underline{C}H_3$).

**2-hydroxy-N-(5-phenyl-1,3,4-oxadiazol-2-yl)acetamide (8)**. A solution of cesium formate (2.18 g, 13 mmol) and 2-oxo-2-((5-phenyl-1,3,4-oxadiazol-2-yl)amino)ethyl acetate (**9**) (1.12 g, 4 mmol) in dry methanol (30 mL) was refluxed in anhydrous conditions for 4 h and monitored by HPLC (gradient of $CH_3OH$ in $H_2O$ from 10 % to 100 % in 30 min, $\lambda$= 200-350 nm, flow= 1 mL/min). The solution was concentrated under reduced pressure and the residue was purified by flash column chromatography on silica gel using a gradient of $CH_3OH$ in

$CH_2Cl_2$ (2% to 10%) as eluent to give the corresponding 2-hydroxy-N-(5-phenyl-1,3,4-oxadiazol-2-yl)acetamide (**8**) as a white powder (0.42 g, 44 %). $R_f$ : 0.36 ($CH_2Cl_2/CH_3OH$ 92:8). $^1H$ NMR (500 MHz, DMSO-$d_6$): $\delta_H$ 10.80 (s, 1H, N$\underline{H}$), 7.88 – 7.85 (m, 2H, C$\underline{H}$-Ph), 7.62 – 7.50 (m, 3H, C$\underline{H}$-Ph), 4.76 (s, 1H, C$\underline{H_2}$OH), 4.73 (s, 1H, C$\underline{H_2}$OH); $^{13}C$ NMR (125 MHz, DMSO-$d_6$): $\delta_C$ 169.92 ($\underline{C}$ONH), 169.12 ($\underline{C}$-2), 159.61 ($\underline{C}$-5), 132.09 ($\underline{C}$H-Ph), 131.99 ($\underline{C}$H-Ph), 128.53 ($\underline{C}$H-Ph), 127.47 ($\underline{C}$H-Ph), 127.42 ($\underline{C}$H-Ph), 127.91 ($\underline{C}$-Ph), 70.77 ($\underline{C}H_2$). HPLC, eluting with 0.1% TFA $H_2O$/MeOH 10:90 to 0/100 in 20 min, 0/100 for 5 min; Flow= 1mL/min, $\lambda$ = 236 nm $t_R$= 9.7 min (98%). HRMS (ESI, negative ion mode): *m/z* calcd 218.0566 $(M - H)^+$, found 218.0576 $(M - H)^+$.

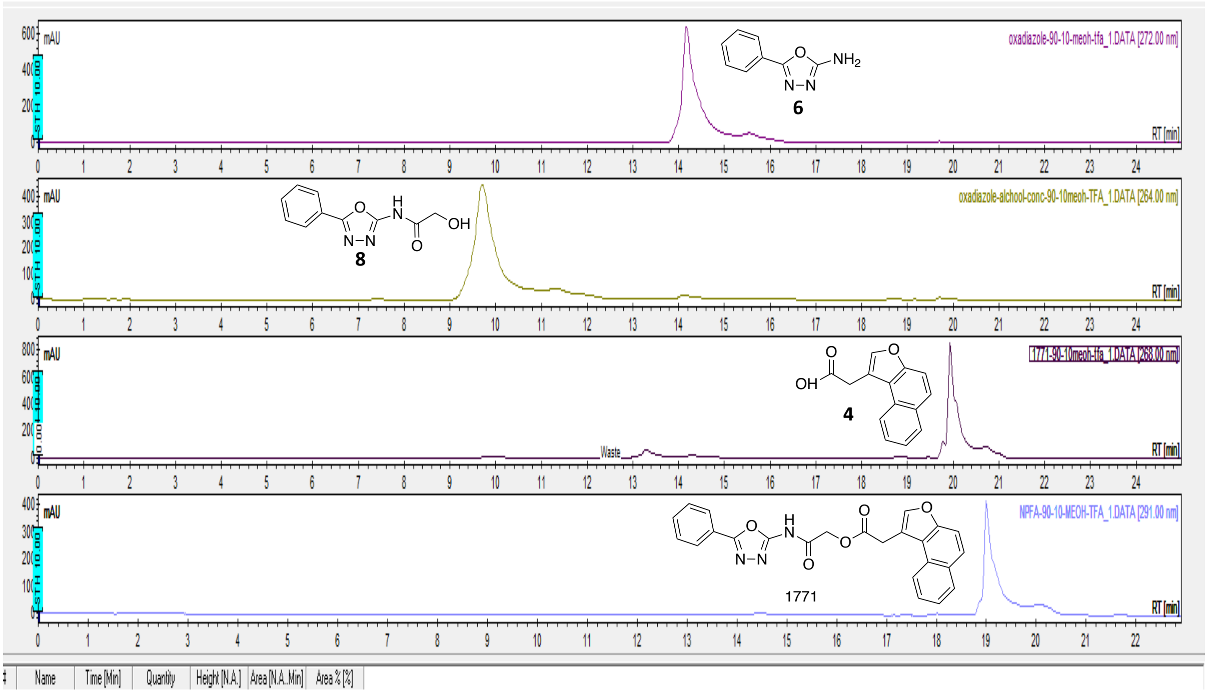

**Figure S1.** Stack of the HPLC traces of compounds **6**, **8**, **4** and 1771. HPLC eluting with 0.1 % TFA $H_2O/CH_3OH$ from 90/10 to 0/100 in 20 min, 0/100 5 min, F= 1 mL/min.

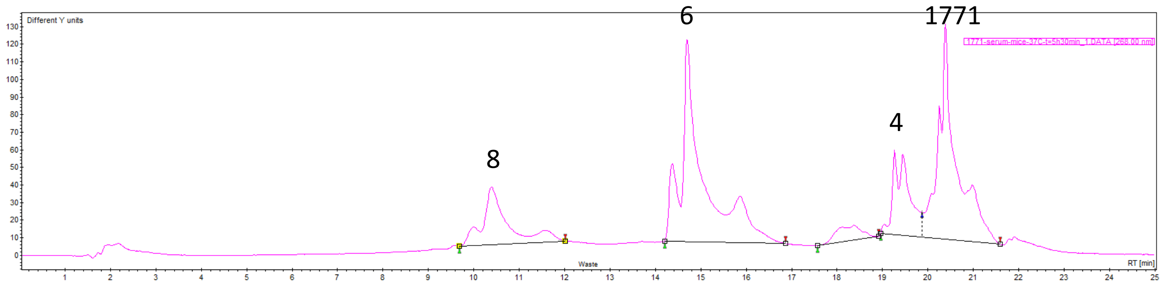


**Figure S2.** Reverse-phase HPLC trace after 5h30 min incubation of 1771 in mouse serum (100 mM phosphate buffer pH 7.4) at 37ºC. HPLC eluting with 0.1 % TFA $H_2O/CH_3OH$ from 90/10 to 0/100 in 20 min, 0/100 5 min, F= 1 mL/min, λ = 268 nm

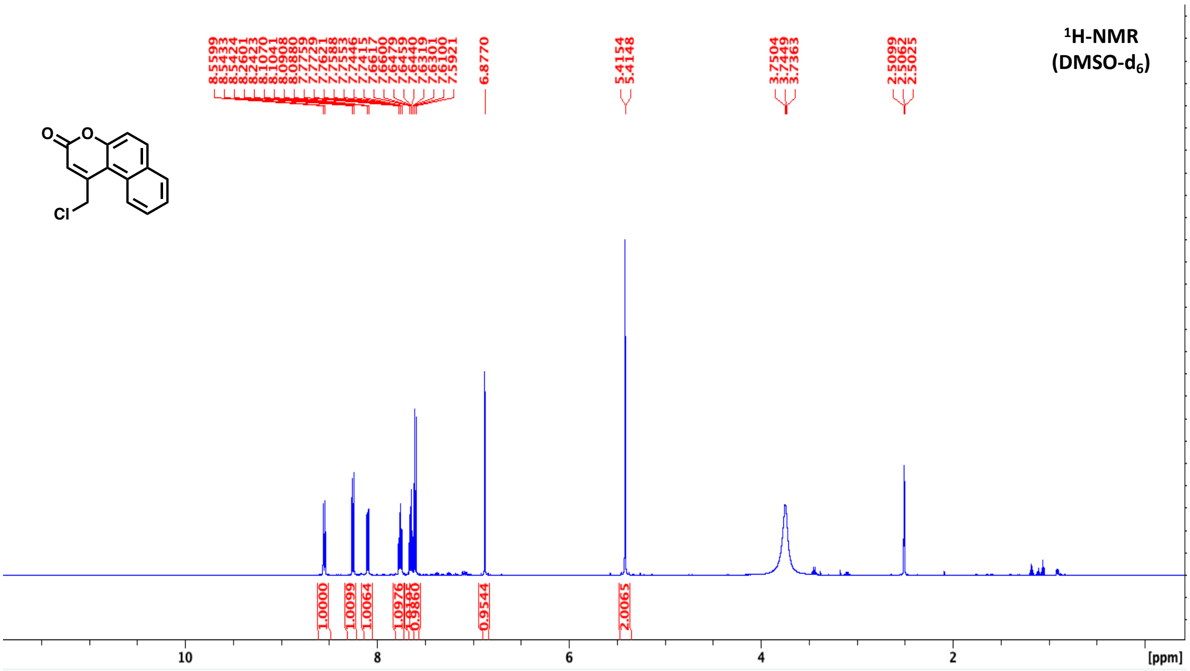


**Figure S3:** $^{1}$H-NMR (500 MHz, DMSO-d$_6$) of compound **3**

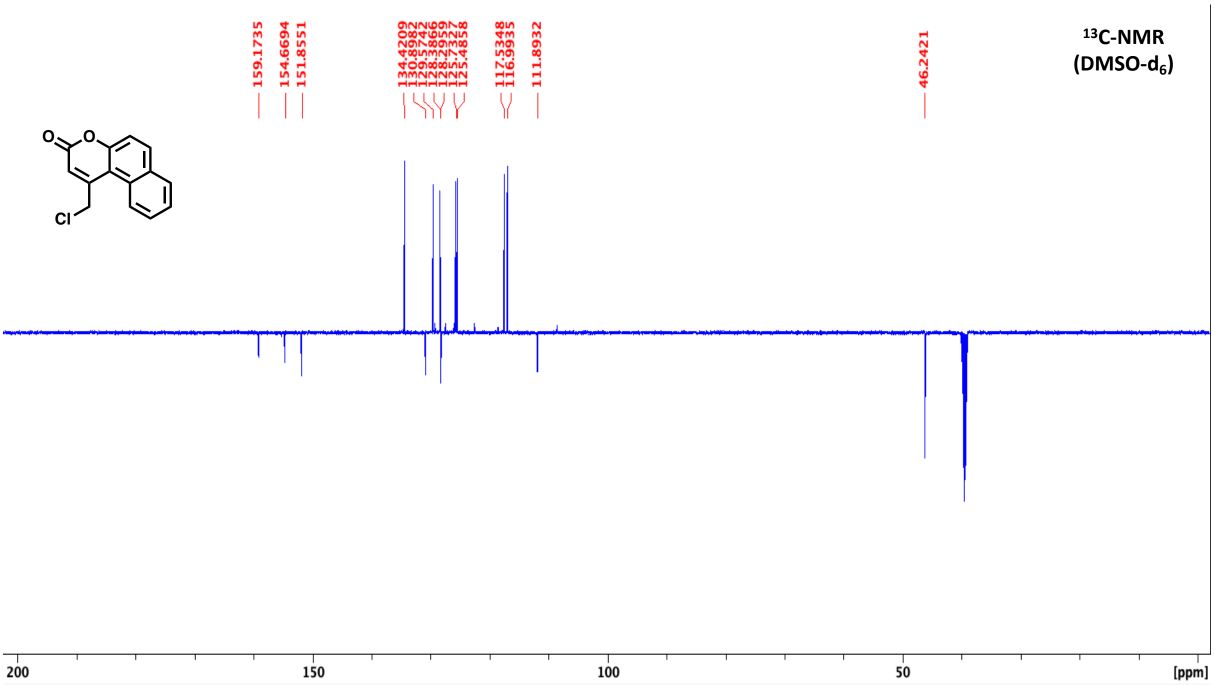


**Figure S4**. $^{13}$C-NMR (500 MHz, DMSO-d$_6$) of compound **3**

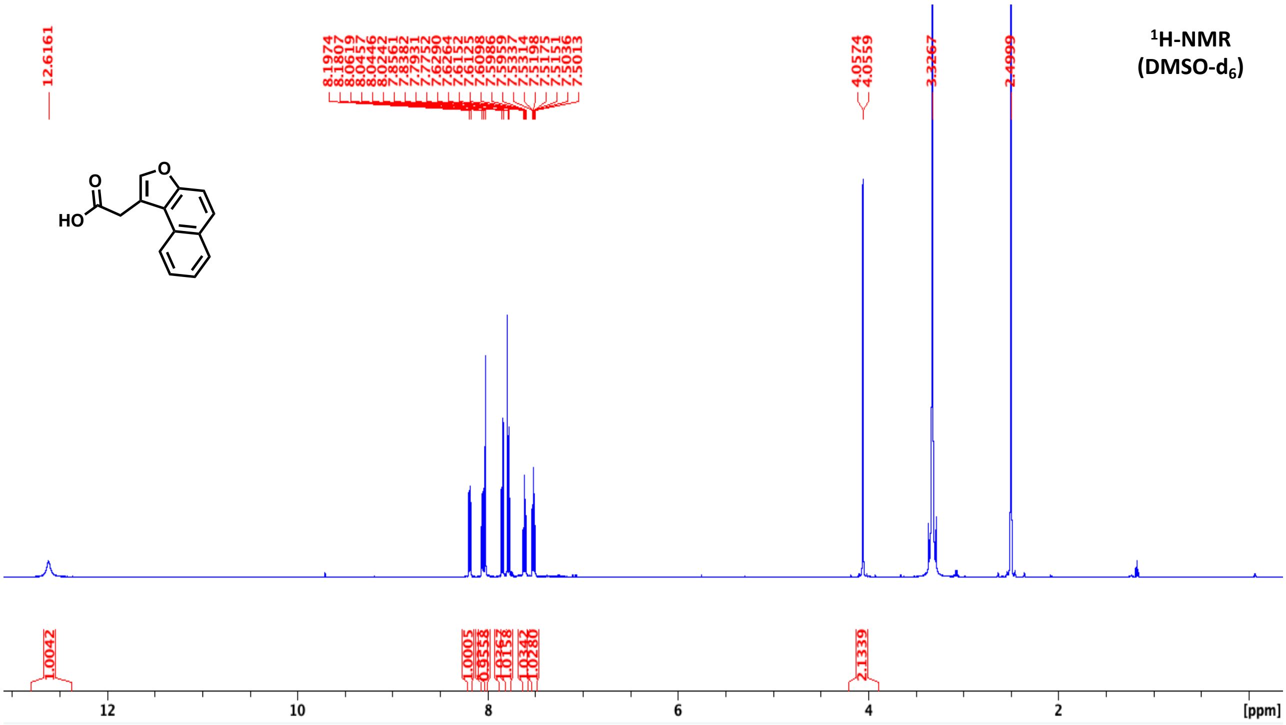


**Figure S5:** $^{1}$H-NMR (500 MHz, DMSO-d$_6$) of compound **4**

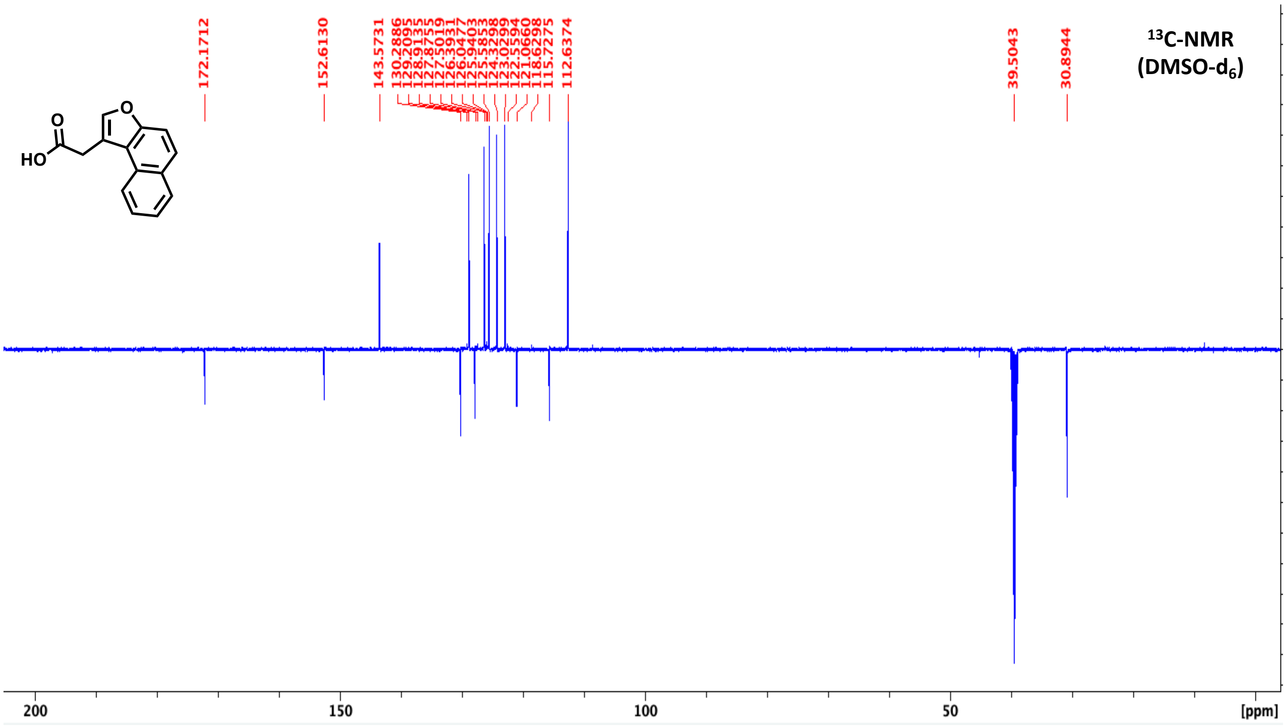


**Figure S6:** $^{13}$C-NMR (500 MHz, DMSO-d$_6$) of compound **4**

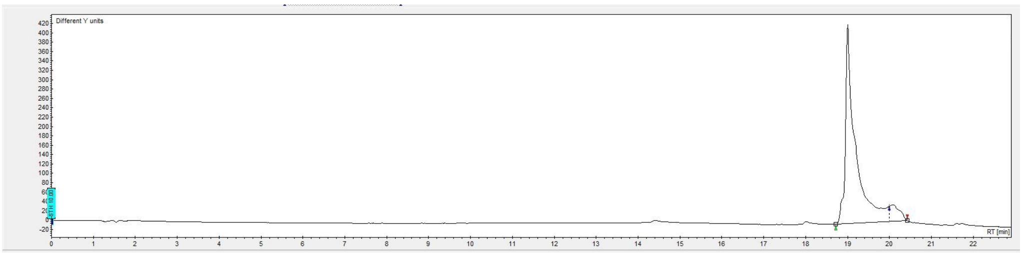


**Figure S7:** HPLC trace of compound **4** (0.1% TFA $H_2O$/MeOH 10:90 to 0/100 in 20 min, 0/100 for 5 min; Flow 1mL/min, $\lambda$= 291 nm)

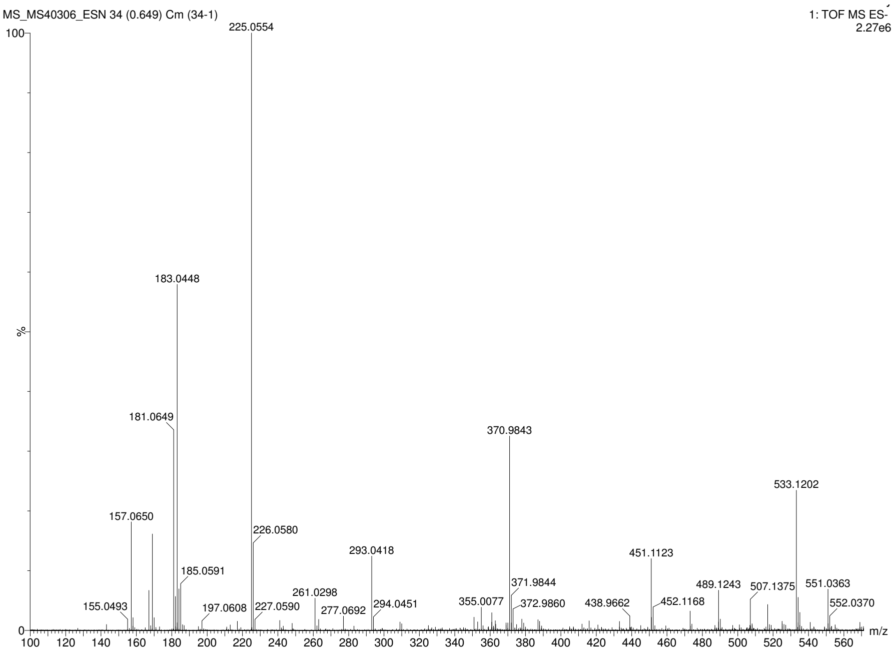


| | | | | | | | | |
|---|---|---|---|---|---|---|---|---|
| Minimum: | | | | -1.5 | | | | |
| Maximum: | | 5.0 | 5.0 | 100.0 | | | | |
| Mass | Calc. Mass | mDa | PPM | DBE | i-FIT | Norm | Conf(%) | Formula |
| 225.0554 | 225.0552 | 0.2 | 0.9 | 10.5 | 528.0 | n/a | n/a | C14 H9 O3 |

**Figure S8:** HRMS of compound **4.**

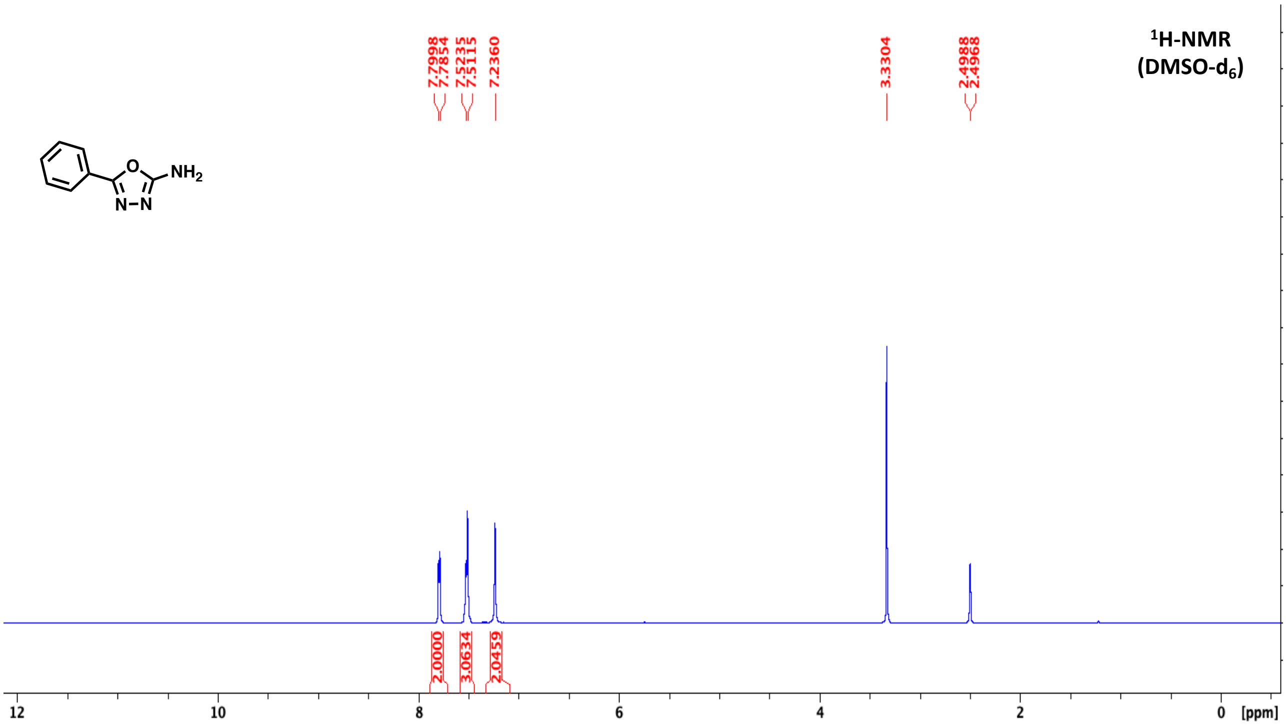


**Figure S9:** $^{1}$H-NMR (500 MHz, DMSO-d$_6$) of compound **6**

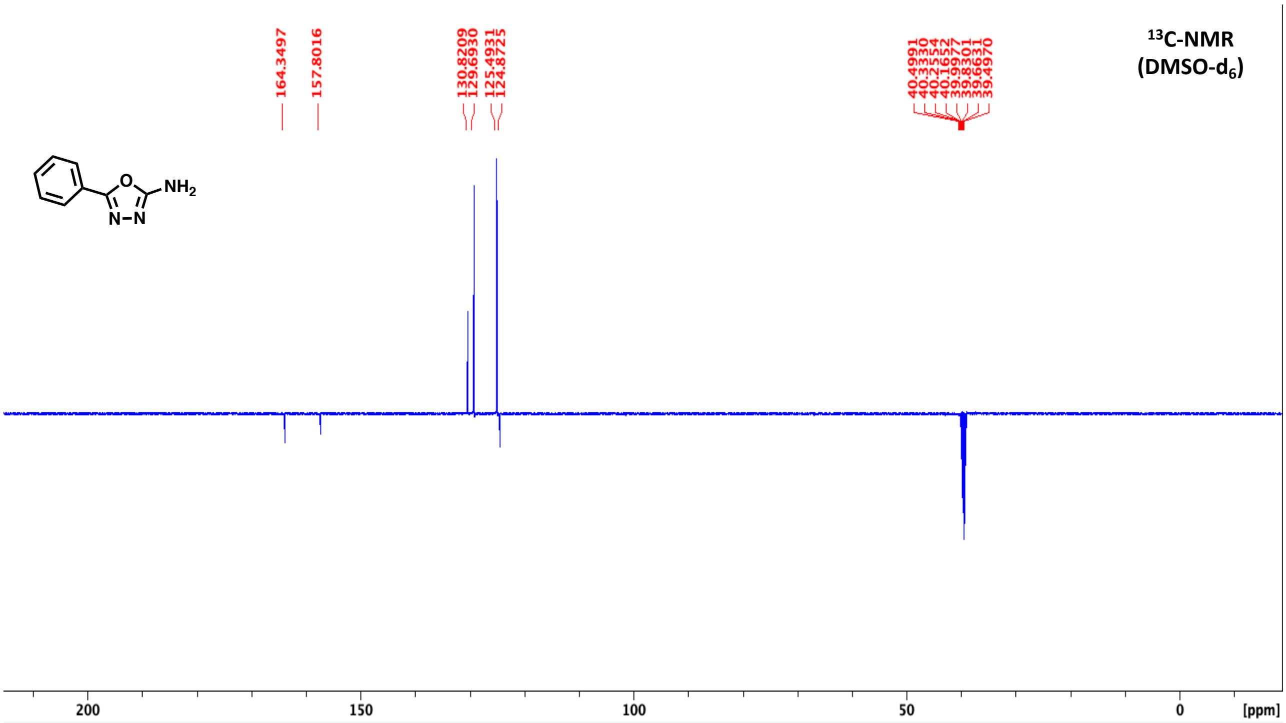


**Figure S10:** $^{13}$C-NMR (500 MHz, DMSO-d$_6$) of compound **6**

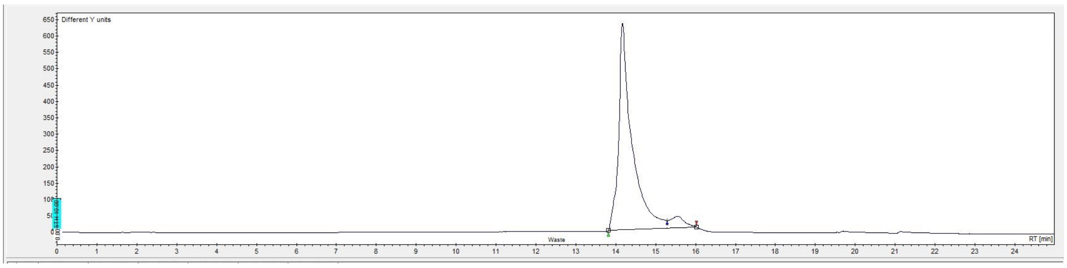

**Figure S11:** HPLC trace of compound **6** (0.1% TFA $H_2O$/MeOH 10:90 to 0/100 in 20 min, 0/100 for 5 min; Flow 1mL/min, $\lambda$= 291 nm)

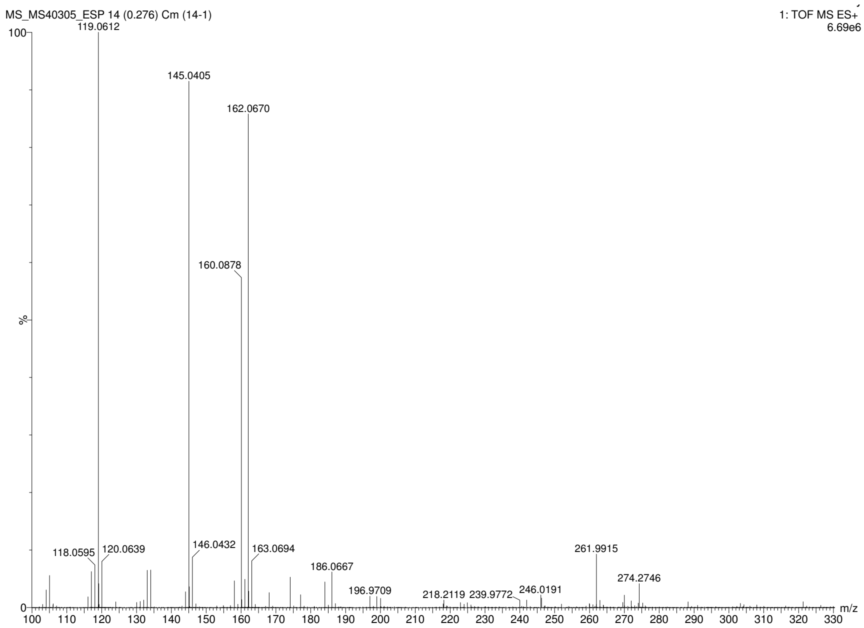


| | | | | | | | | |
|---|---|---|---|---|---|---|---|---|
| Minimum: | | | | -1.5 | | | | |
| Maximum: | | 5.0 | 5.0 | 100.0 | | | | |
| Mass | Calc. Mass | mDa | PPM | DBE | i-FIT | Norm | Conf(%) | Formula |
| 162.0670 | 162.0667 | 0.3 | 1.9 | 6.5 | 570.5 | n/a | n/a | C8 H8 N3 O |

**Figure S12:** HRMS of compound **6**

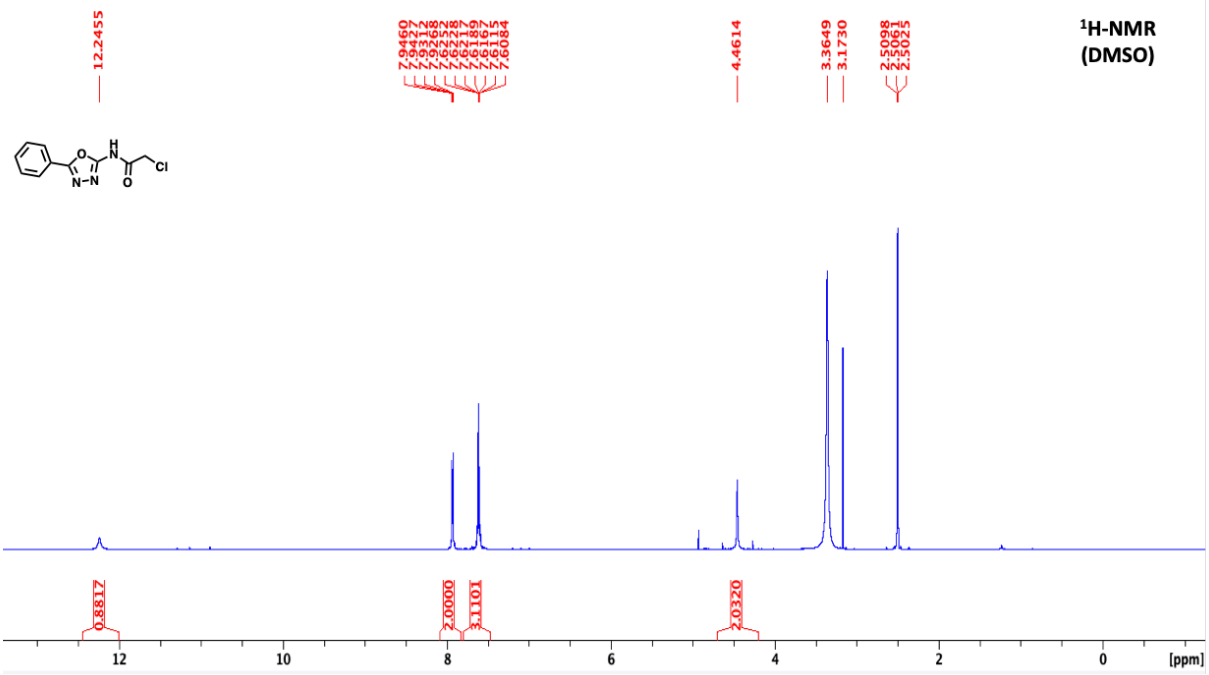


**Figure S13:** $^{1}$H-NMR (500 MHz, DMSO-$d_6$) of compound **7**

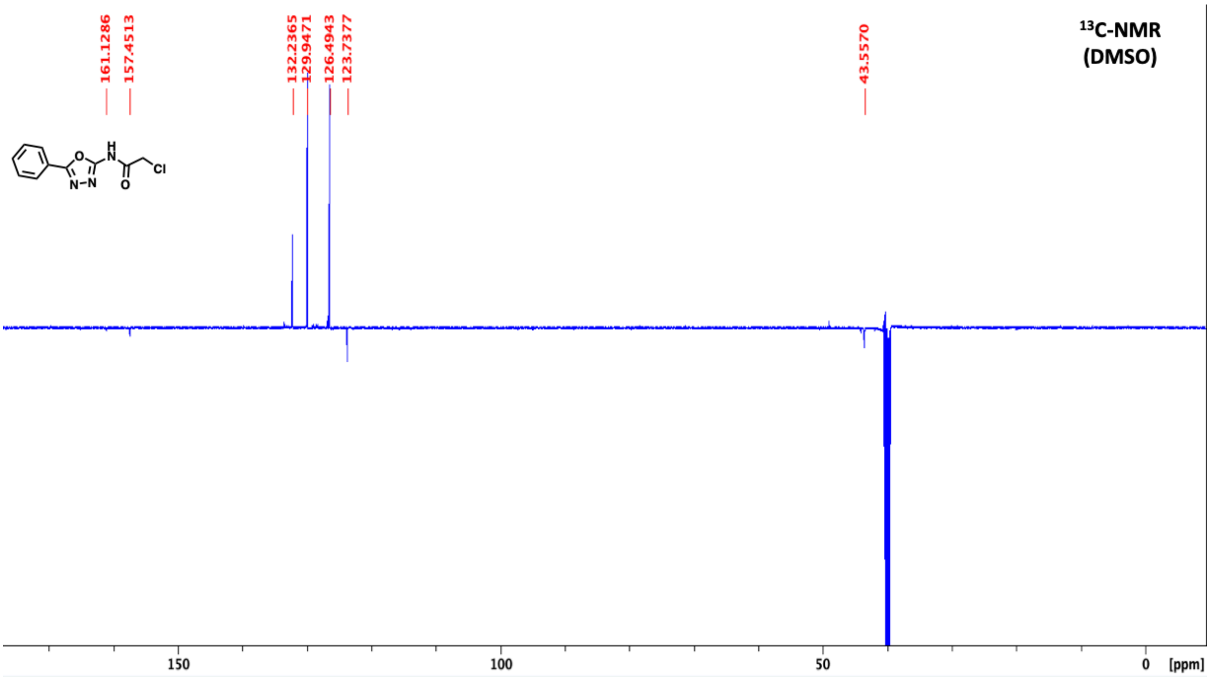


**Figure S14:** $^{13}$C-NMR (500 MHz, DMSO-$d_6$) of compound **7**

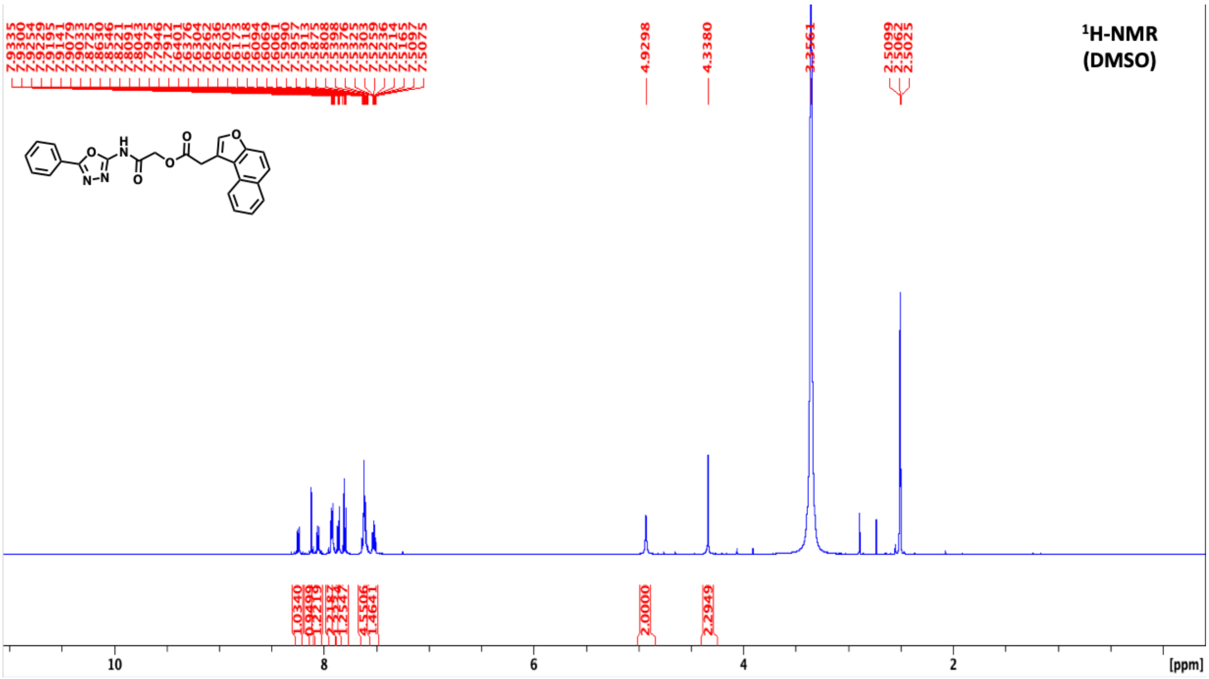


**Figure S15:** $^{1}$H-NMR (500 MHz, DMSO-$d_6$) of compound **1**

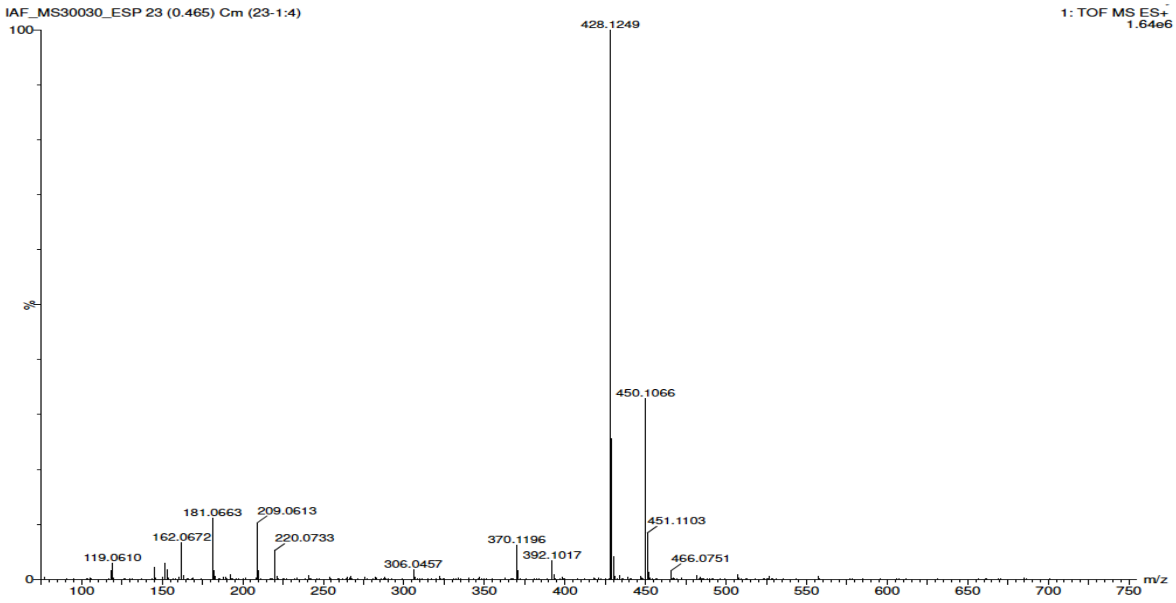


| Mass | Calc. Mass | mDa | PPM | DBE | i-FIT | Norm | Conf(%) | Formula |
|---|---|---|---|---|---|---|---|---|
| 428.1249 | 428.1246 | 0.3 | 0.7 | 17.5 | 236.9 | n/a | n/a | C24 H18 N3 O5 |

**Figure S16:** HRMS of compound **1** (1771)

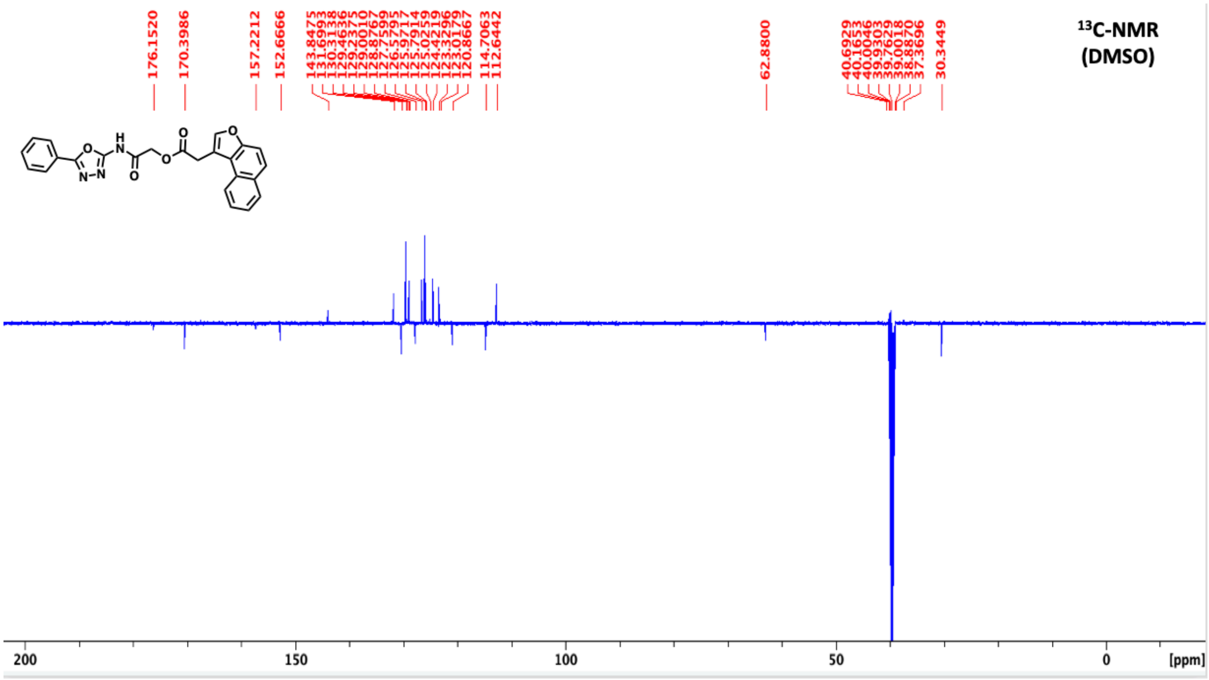


**Figure S17:** $^{13}$C-NMR (500 MHz, DMSO-$d_6$) of compound **1**

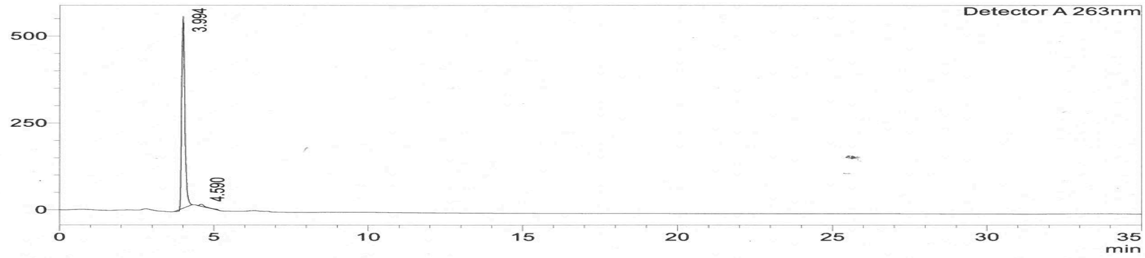


**Figure S18:** HPLC trace of compound 1 ($H_2O$/ACN 40:60 for 25 min; 60 to 100 in 5 min, 100 for 5 min; Flow 1mL/min, λ=263 nm)

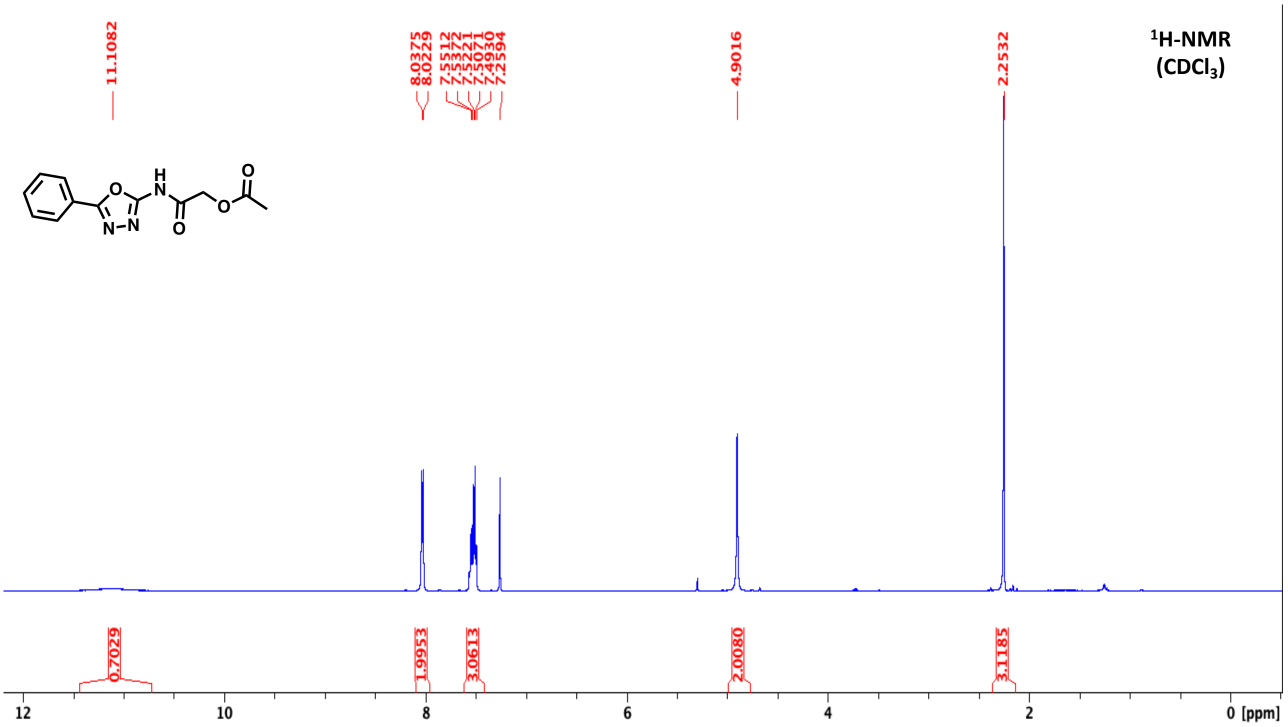


**Figure S19:** $^{1}$H-NMR (500 MHz, DMSO-d$_6$) of compound **9**

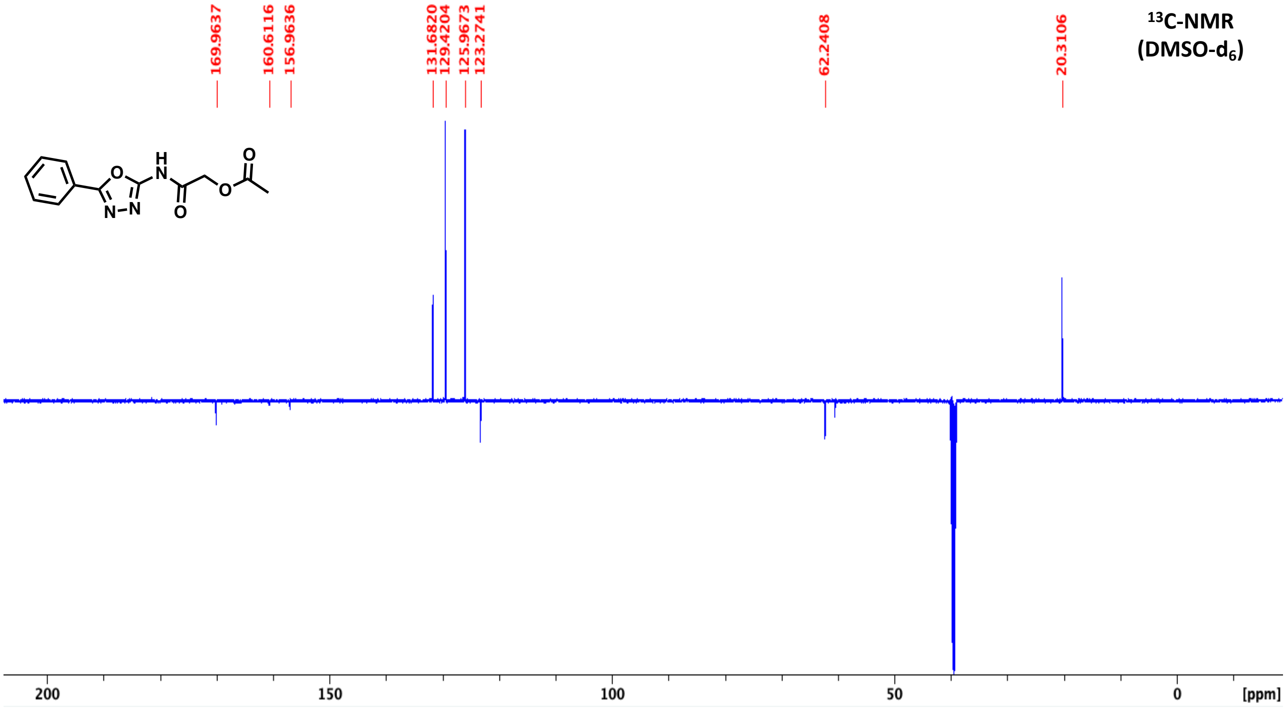


**Figure S20:** $^{13}$C-NMR (500 MHz, DMSO-d$_6$) of compound **9**

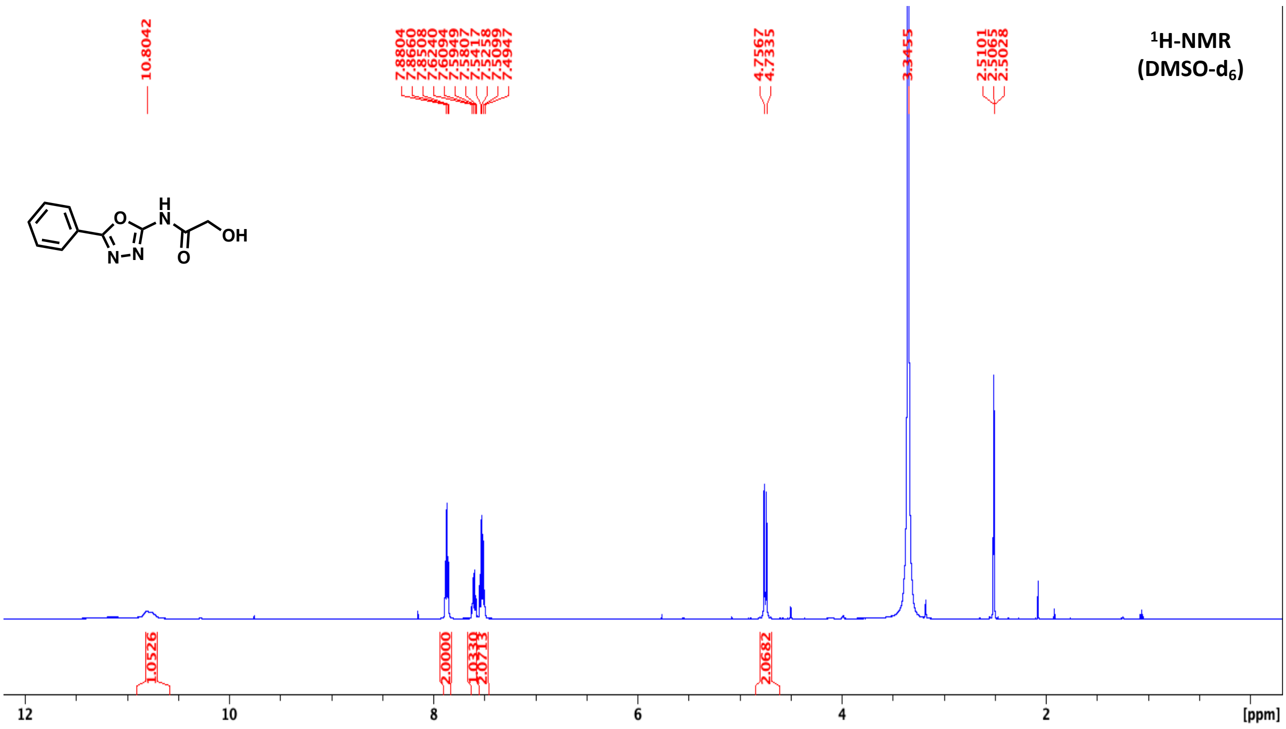


**Figure S21:** $^{1}$H-NMR (500 MHz, DMSO-d$_6$) of compound **8**

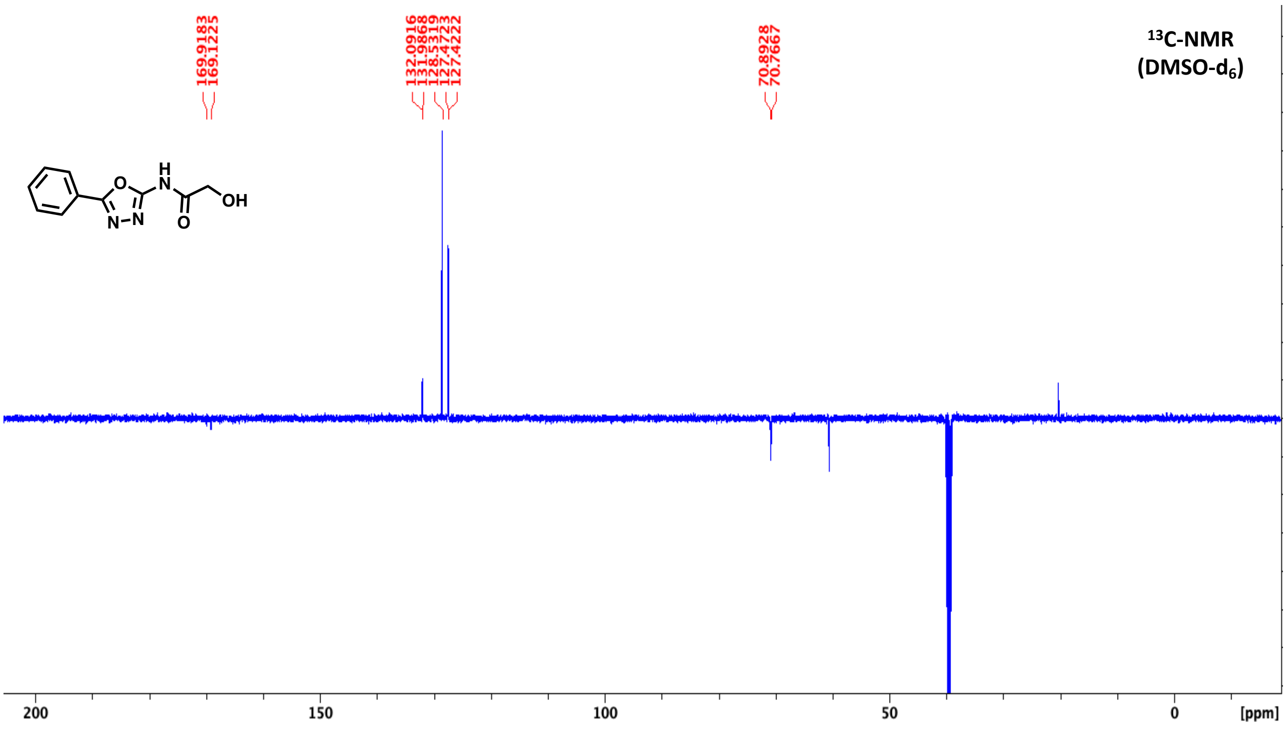


**Figure S22:** $^{13}$C-NMR (500 MHz, DMSO-d$_6$) of compound **8**

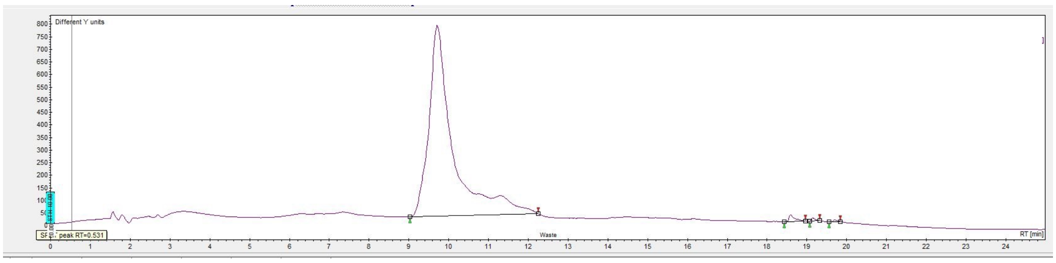

**Figure S23:** HPLC trace of compound **8** (0.1% TFA $H_2O$ :MeOH 10:90 to 0/100 in 20 min, 0/100 for 5 min; Flow 1mL/min, $\lambda$= 236 nm)

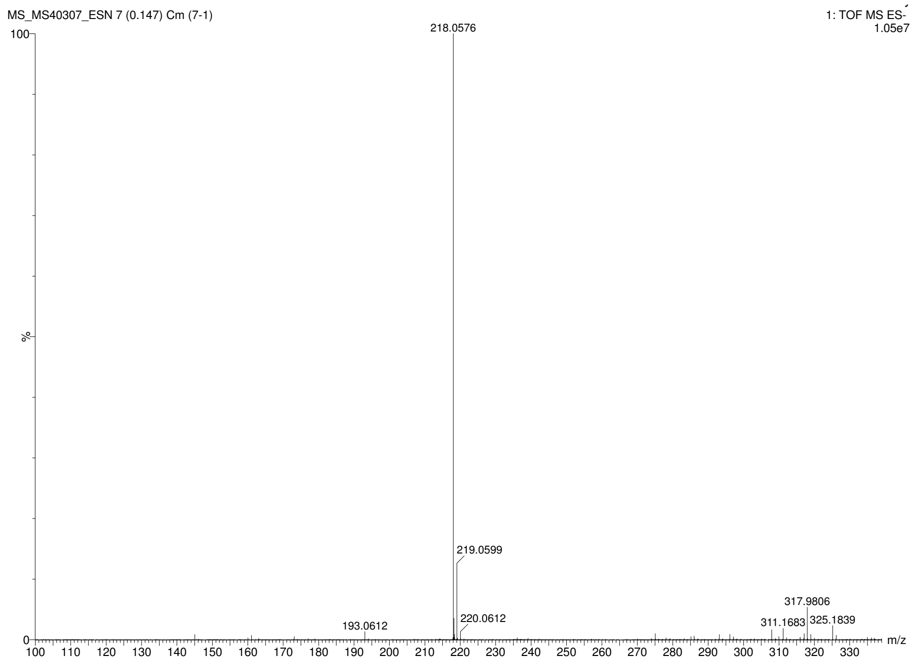


| | Mass | Calc. Mass | mDa | PPM | DBE | i-FIT | Norm | Conf(%) | Formula |
|---|---|---|---|---|---|---|---|---|---|
| Minimum: | | | | | -1.5 | | | | |
| Maximum: | | | 5.0 | 5.0 | 100.0 | | | | |
| | 218.0576 | 218.0566 | 1.0 | 4.6 | 8.5 | 799.8 | n/a | n/a | C10 H8 N3 O3 |

**Figure S24:** HRMS of compound **8**
